# Unveiling DEFB1 as a novel driver and promising therapeutic target in lung adenocarcinoma

**DOI:** 10.1038/s41419-026-08748-4

**Published:** 2026-04-20

**Authors:** Jiaqi Liang, Yanjun Yi, Junkan Zhu, Huan Zhang, Yidu Hu, Yang Lu, Lewei Duan, Guoshu Bi, Yunyi Bian, Xiaodong Yang, Guangyao Shan, Zongwu Lin, Qun Wang, Cheng Zhan, Wei Jiang

**Affiliations:** 1 Department of Thoracic Surgery, Zhongshan Hospital, Fudan University, Shanghai, PR China; 2 Cancer Center, Zhongshan Hospital, Fudan University, Shanghai, PR China; 3 Department of Thoracic Surgery, Sichuan Clinical Research Center for Cancer, Sichuan Cancer Hospital & Institute, Sichuan Cancer Center, University of Electronic Science and Technology of China, Chengdu, PR China; 4 Department of General Practice, Danzhou Public Security Supervision Hospital, Danzhou, Hainan PR China; 5 Department of Thoracic Surgery, Shanghai Chest Hospital, Shanghai Jiao Tong University, Shanghai, PR China; 6 Department of Thoracic Surgery, Shanghai Pulmonary Hospital, Tongji University, Shanghai, PR China; 7 Department of Thoracic Surgery, Shanghai Geriatric Medicine Center, Shanghai, PR China

**Keywords:** Non-small-cell lung cancer, Cell growth

## Abstract

Lung adenocarcinoma, one of the most prevalent malignancies, underscores the urgency of identifying genes linked to its proliferation and prognosis for developing targeted therapies. In this study, we performed genome-wide CRISPR/Cas9 screening both in vitro and in vivo, and subsequently cross-referenced the findings with prognosis-related genes in lung adenocarcinoma. The screening results revealed that DEFB1 promotes lung adenocarcinoma proliferation, while our integrated analysis of single-cell sequencing, multiplex immunohistochemistry, TCGA, and GEO data concurrently demonstrated elevated DEFB1 expression in cancer cells, along with a negative correlation between DEFB1 expression and patient survival. Subsequently, functional studies employing DEFB1 knockout cells, re-expressed DEFB1 cells, and knockout cells supplemented with exogenous DEFB1 revealed that DEFB1 significantly enhances cell proliferation, migration, and invasion. Co-immunoprecipitation combined with mass spectrometry experiments was performed to uncover the mechanism of DEFB1, demonstrating that it interacts with Periplakin (PPL) to induce epithelial-to-mesenchymal transition (EMT) and proliferation, while simultaneously binding to Macrophage Migration Inhibitory Factor (MIF) to enhance M2 macrophage polarization. Furthermore, we developed multiple anti-DEFB1 monoclonal antibodies and found one of them, mAb-5, potently blocked DEFB1’s function and inhibited lung adenocarcinoma progression in cell lines, organoids, xenografts, and spontaneous lung cancer models, while maintaining a favorable safety profile. Overall, our study identifies DEFB1 as a novel driver of lung adenocarcinoma, and the anti-DEFB1 monoclonal antibody mAb-5 emerges as a promising therapeutic candidate with significant potential.

## Introduction

Lung cancer ranks among the most common and deadly cancers worldwide [1, 2]. Among its diverse pathological subtypes, lung adenocarcinoma has emerged as the leading one, accounting for over 60% of newly diagnosed lung cancer cases each year [2, 3]. Characterized by rapid proliferation, high invasiveness, and a strong tendency for metastasis, lung adenocarcinoma poses a significant threat to patients’ lives and overall well-being [4–6].

Despite significant progress made by conventional treatment modalities, including surgery, targeted therapy, chemotherapy, and radiotherapy, in extending the survival of lung adenocarcinoma patients, their efficacy often leaves much to be desired for those with advanced-stage disease, particularly those lacking specific gene mutations [4]. This treatment gap highlights the pressing need to delve deeper into the molecular mechanisms underlying lung adenocarcinoma proliferation and to discover novel therapeutic targets and strategies.

To address this critical challenge, our study employed a genome-wide knockout screening [7, 8] for the systematic and comprehensive evaluation of the impact of each gene knockout on lung adenocarcinoma cell proliferation. By conducting high-throughput screening in both in vitro and in vivo, coupled with single-cell sequencing, we identified defensin beta 1 (DEFB1) as a gene of particular interest.

DEFB1, encoding an antimicrobial peptide produced by epithelial cells that belongs to the defensin family, plays a crucial role in safeguarding mucosal surfaces from microbial assaults [9, 10]. However, despite numerous studies reporting significant alterations in DEFB1 expression across various epithelial-derived tumors, its functions within tumors, including lung cancer, remain largely enigmatic [11–13]. In this study, we elucidated, for the first time, the oncogenic roles and underlying mechanisms of DEFB1 in lung adenocarcinoma, thereby advancing novel insights and identifying potential therapeutic targets for this devastating disease.

## Materials and methods

### Cell line sources and culture conditions

The human lung adenocarcinoma cell lines A549 and PC-9, human embryonic kidney cell line 293T, and human monocytic leukemia cell line THP-1 were obtained from the Cell Bank of the Chinese Academy of Sciences (https://www.cellbank.org.cn). A549, PC-9, and 293T cells were cultured in high-glucose Dulbecco’s Modified Eagle’s Medium (DMEM) (KeyGen Biotech, Nanjing, Jiangsu, China) supplemented with 10% fetal bovine serum (Evergreen, Hangzhou, Zhejiang, China) and 100 U/mL penicillin/streptomycin (Sangon Biotech, Shanghai, China) at 37 °C in a 5% CO₂ incubator. THP-1 cells were cultured in antibiotic-free RPMI-1640 media (KeyGEN) supplemented with 10% fetal bovine serum (Evergreen) at 37 °C in a 5% CO₂ incubator. The cell lines were subcultured every five to seven days based on their proliferation rates. To prevent mycoplasma contamination, the cells were tested every two months using a PCR-based method, as recommended in the literature [14, 15]. Only cells that tested negative for mycoplasma were used in subsequent experiments.

### DEFB1 and 3× Flag-tagged DEFB1 proteins

Recombinant human DEFB1 protein was obtained from Abcam (#ab243114, Cambridge, UK). To obtain 3× Flag-tagged DEFB1 protein, a plasmid encoding human DEFB1 with a C-terminal 3× Flag tag was constructed by GeneChem (Shanghai, China) and transfected into 293T cells using Lipo8000™ Transfection Reagent (Beyotime, Shanghai, China). Subsequently, the 3× Flag-tagged DEFB1 protein in the cell culture supernatant was purified using Anti-Flag Affinity Gel (Secretory Protein) (Yeasen Biotechnology, Shanghai, China). The protein concentration was quantified using a BCA Protein Quantification Kit (Yeasen Biotechnology), and purity was analyzed by sodium dodecyl sulfate-polyacrylamide gel electrophoresis (SDS-PAGE). In relevant experiments, DEFB1 or 3× Flag-tagged DEFB1 proteins were exogenously added to the culture medium at a concentration of 200 pg/mL. Additionally, the culture medium was refreshed every three days to maintain the protein concentration.

### CRISPR/Cas9 genome-wide knockout screening, gene knockout, and overexpression

As previously reported [8], this study employed a lentiviral genome-scale human CRISPR knockout pooled library (GeCKO v2) (https://www.addgene.org/pooled-library/zhang-human-gecko-v2/) (GeneChem) for genome-wide knockout screening experiments. The library contained 123,411 unique single-guide RNA (sgRNA) sequences targeting 19,050 human genes (approximately 6 sgRNAs per gene on average, along with 2000 control sgRNAs) [16].

Briefly, the library was transfected into the A549 cell line, and puromycin (Beyotime) was used for selection. In in vitro experiments, the transfected cells were cultured for 14 days. In in vivo experiments, the transfected cells were subcutaneously transplanted into the right flanks of six male BALB/c nude mice (Shanghai Model Organisms Center) at a dose of 2 × 10^6^ cells per mouse, and the mice were maintained for four weeks. Subsequently, genomic DNA was extracted from the cultured cells and tumor xenografts, and the sgRNAs were sequenced by GeneChem. If an sgRNA led to reduced tumor proliferation in vitro or in vivo, its final frequency would significantly decrease.

Meanwhile, this study utilized the CRISPR/Cas9 technology to knock out the DEFB1 and periplakin (PPL) genes in cell lines. Briefly, the sgRNAs were cloned into plasmids containing a puromycin resistance gene and the hSpCas9 gene, and the edited vectors were then packaged into lentiviruses by GeneChem. Then, cells were transfected with lentiviruses according to the protocol provided by GeneChem. Seventy-two hours after infection, the cells were cultured in a selection medium containing 5 μg/mL puromycin (Beyotime) for 48 h. The sgRNA sequence for DEFB1 knockout is 5′-CATTACAATTGCGTCAGCAG-3′, while that for PPL knockout is 5′-GTACATGGAGGACTACCACC-3′.

For DEFB1 re-overexpression experiments, a sgRNA-resistant DEFB1 overexpression lentivirus with a geneticin resistance gene was generated (GeneChem) by introducing mutations in the sgRNA target sequence and the following protospacer adjacent motif (5′-CACTATAACTGTGTTAGTAGCGGT-3′) to disrupt sgRNA binding while preserving the wild-type protein sequence. Then the lentivirus was transfected into DEFB1 knockout cells, and stable re-overexpressing cell lines were established by geneticin selection.

### TCGA survival analysis

Gene expression, clinical, and survival data of lung adenocarcinoma samples of The Cancer Genome Atlas (TCGA) were obtained from the UCSC Xena website (https://gdc.xenahubs.net) [17]. Frozen samples of lung adenocarcinoma were included (with “-01A” suffix), while recurrent tumor samples (with “-02A” suffix), paraffin-embedded samples (with “-01B” or “-11B” suffix) were excluded. The fragments per kilobase of exon model per million mapped fragment (FPKM) data of both datasets were log2 (FPKM+1) transformed before being analyzed.

Gene expression-based survival analysis was conducted using the survminer package in R software (Version 4.3.1). For each gene, the optimal expression cutoff was determined using maximally selected rank statistics (surv_cutpoint), dividing patients into high/low expression subgroups. Cox proportional hazards models were then fitted to these subgroups to calculate hazard ratios (HR) and 95% confidence intervals (CI), with try-catch error handling to exclude genes with non-convergent models from final results.

### Multiplex immunohistochemistry

The tissue specimens were obtained from patients who underwent surgery at the Department of Thoracic Surgery, Zhongshan Hospital, Fudan University, and were diagnosed with lung adenocarcinoma, or from tumor xenografts and primary lung tumors formed in nude mice in this study.

As previously described [18], each tissue section underwent the following processing steps in sequence: paraffin dissolution, xylene dewaxing, hydration, antigen retrieval, cooling, permeabilization, and blocking. Subsequently, the sections were incubated with primary antibodies overnight at 4 °C, followed by washing with phosphate-buffered saline (PBS) to remove unbound antibodies. Next, the sections were incubated with corresponding secondary antibodies for 1 h and then treated with tyramide signal amplification (TSA) reagents (Servicebio, Wuhan, Hubei, China) for 10 min at room temperature.

To remove the primary and secondary antibodies that had bound to the tissues, the sections were placed in citrate antigen retrieval buffer (pH = 6.0) and heated in a microwave oven until boiling for 10 min. This blocking step was repeated once, followed by reapplication of the next primary antibody, subsequent secondary antibody incubation, and TSA reagent treatment. For nuclear staining, the slides were stained with 4′,6-diamidino-2-phenylindole (DAPI; Servicebio). The TSA reagents used were as follows: iF488-TSA (1:500, Servicebio), iF546-TSA (1:500, Servicebio), iF647-TSA (1:500, Servicebio), and iF700-TSA (1:500, Servicebio). The primary antibodies used in this section are listed in Supplementary Table 1.

Fluorescence intensity profiles along selected line regions were quantified using ImageJ image analysis software (version 1.54g) (https://imagej.net/ij). Pearson’s correlation coefficients were calculated based on the fluorescence intensity values of the corresponding fluorescence channels.

### High-content imaging analysis

To investigate cell proliferation characteristics under different treatment conditions, A549 and PC-9 cells transfected with an mCherry fluorescent marker from different treatment groups were uniformly seeded in 24-well plates at a density of 1 × 10^4^ cells per well. After seeding, the 24-well plates were incubated in a cell culture incubator for 24 h to ensure proper cell adhesion. Subsequently, dynamic monitoring of the cells was performed using the Operetta CLS High-Content Imaging Analysis System (PerkinElmer, USA). The excitation wavelength was set at 587 nm, and the emission wavelength for detection was 660 nm. Starting from 24 h after cell seeding and spanning a time period of 120 h, continuous image acquisition was carried out at 6-h intervals.

After image acquisition, the obtained images were processed and analyzed using ImageJ to accurately identify and calculate the area of the red fluorescent region in each field of view. This area was used as a key indicator for measuring cell proliferation. Six fields of view were randomly selected from each well for fluorescence area measurement, and the mean values were calculated for subsequent data statistics and comparative analysis.

### Alama blue assay

Cells were plated at a density of 1000 cells per well in 96-well plates. After incubation for 24, 48, 72, 96, and 120 h, 10 μL of Alamar blue reagent (Yeasen Biotechnology) was added to each respective well, followed by a 2-h incubation at 37 °C with 5% CO₂. Fluorescence was measured using a SpectraMax iD5 Multi-Mode Microplate Reader (Molecular Devices, USA) with an excitation wavelength of 545 nm and an emission wavelength of 590 nm.

### Transwell assay for cell migration and invasion

As previously reported [19, 20], for the migration assay, 1 × 10^4^ logarithmic-phase cells were resuspended in serum-free medium and seeded into the upper chambers of Transwell inserts (8.0 μm pore size, Corning, USA). For invasion assay, Matrigel matrix gel (Yeasen Biotechnology) was diluted 1:5 with serum-free medium and evenly spread onto the upper chamber membranes of the Transwell inserts (8.0 μm pore size, Corning). The inserts were incubated at 37 °C for two hours to allow gel solidification. Then, logarithmic-phase cells (1 × 10^4^) were resuspended in serum-free medium and seeded into the upper chambers.

The lower chambers were filled with culture medium containing 10% FBS (Evergreen). After 24 h of culture, the medium was discarded. The residual cells on the upper surfaces of the Transwell chambers were gently removed using cotton swabs. The cells on the lower surfaces of the upper chambers were fixed with 4% paraformaldehyde for 15 min and subsequently rinsed twice with PBS. They were then stained with 0.1% crystal violet for 10 min and washed with distilled water until the background was free of residual stain. Five fields of view were randomly selected under a 100× microscope, and the number of cells that had migrated to the lower chambers was counted.

### RNA-sequencing (RNA-Seq)

In the RNA-Seq experiment, total RNA was extracted using TRNzol Universal Reagent (TIANGEN, Beijing, China). The purity and concentration of the RNA were then assessed using a NanoDrop 2000 spectrophotometer (Thermo Fisher, Waltham, MA, USA). Additionally, the integrity of the RNA was evaluated using an Agilent 2100 Bioanalyzer (Agilent Technologies, Santa Clara, CA, USAUSA). Transcriptome libraries were constructed using the VAHTS Universal V10 RNA-seq Library Prep Kit (Vazyme Biotech, Nanjing, China) following the provided protocol. The libraries were then sequenced on the NovaSeq 6000 sequencing platform (Illumina, San Diego, CA, USA) using a paired-end 150 bp sequencing strategy by OBiO Tech (Shanghai, China). The differentially expressed genes were identified with a threshold of fold-change >1.5 or <0.667, as well as adjusted *p*-value < 0.05 using the “limma” package of the R software (version 4.5.1) [21]. Then the “clusterProfiler” and the “org.Hs.eg.db” packages of the R software were used to analyze the enriched Gene Ontology (GO) biological processes [22].

### Quantitative real-time PCR (qRT-PCR)

Total RNA was extracted from the cells using TRNzol Universal Reagent (TIANGEN) and reverse-transcribed into cDNA using the Hifair® II 1st Strand cDNA Synthesis Kit (gDNA digester plus) (Yeasen Biotechnology). Subsequently, qPCR amplification was performed on an ABI QuantStudio 5 Real-Time PCR System (Thermo Fisher) using the Hifair® III One Step RT-qPCR SYBR Green Kit (Yensen). Each group contained at least three samples, and each sample was tested in triplicate. The threshold cycle (Ct) values of each gene were normalized to the Ct value of the endogenous control β-actin, and quantitative analysis was performed using the 2^(−ΔΔCt) method. The primers used were synthesized by Sangon Biotech, and their specific sequences are as follows: CD86: F: 5′-CTGCTCATCTATACACGGTTACC-3′; R: 5′-GGAAACGTCGTACAGTTCTGTG-3′; CD206: F: 5′-TCCGGGTGCTGTTCTCCTA-3′; R: 5′-CCAGTCTGTTTTTGATGGCACT-3′; DEFB1: F: 5′-AGTCGCCATGAGAACTTCCTACCT-3′; R: 5′-GACATTGCCCTCCACTGCTGAC-3′; β-actin: F: 5′-TGACGTGGACATCCGCAAAG-3′; R: 5′-CTGGAAGGTGGACAGCGAGG-3′.

### Western blot assay

The cells were lysed using RIPA buffer (Beyotime) containing a protease and phosphatase inhibitor cocktail (TargetMol), and the protein concentration was determined using a BCA Protein Quantification Kit (Yensen). The protein samples were boiled in 5× SDS-PAGE loading buffer (Beyotime) at 100 °C for 10 min. Twenty μg of proteins were separated by SDS-PAGE and transferred onto polyvinylidene fluoride (PVDF) membranes (Merck-Millipore, USA). After blocking the membranes with 5% non-fat milk, they were incubated with specific primary antibodies overnight at 4 °C. The next day, the membranes were washed with Tris-buffered saline-Tween solution, followed by incubation with corresponding secondary antibodies at room temperature. Finally, the protein bands were detected using the BeyoECL Moon Ultra-Sensitive ECL Chemiluminescence Kit (Beyotime). The antibodies were obtained from Abcam (Cambridge, UK), Abmart (Shanghai, China), Abways (Shanghai, China), Beyotime, and Servicebio, with the details including the dilution ratio listed in Supplementary Table S1. Full and uncropped Western blot images are provided in the Supplementary Materials.

### Enzyme-linked immunosorbent assay (ELISA)

The content of DEFB1 in the culture supernatant was determined using the DEFB1 ELISA kit provided by NeoBioscience Technology (Shenzhen, China), according to the manufacturer’s instructions. In brief, 100 μL of the samples and standard solutions were introduced into the assay plate and incubated for a duration of 2 h. Following a thorough washing step, a biotin-conjugated DEFB1 antibody in the ELISA kit was added and incubated for an additional 30 min. Subsequently, horseradish peroxidase (HRP)-labeled streptavidin, the 3,3′,5,5′-Tetramethylbenzidine (TMB) working solution, and the stop solution in the ELISA kit were added in a sequential manner. Finally, the absorbance of the resulting solution was measured using a SpectraMax iD5 Multi-Mode Microplate Reader at 450 nm, and the concentration of DEFB1 in the samples was calculated based on the absorbance values of the standard solutions.

### CoIP/MS and CoIP/Western blot

As previously reported [23, 24], 1 × 10^7^ cells were washed twice with pre-chilled phosphate-buffered saline (PBS) and then lysed with an appropriate amount of NP-40 lysis buffer (Beyotime) containing protease and phosphatase inhibitors (TargetMol). The lysis buffer consisted of 50 mM Tris-HCl (pH 7.4), 150 mM NaCl, and 1% NP-40. The lysates were sonicated to break the cells and release the proteins, followed by centrifugation at 12,000 × *g* for 5 min at 4 °C. The supernatants were collected and stored at −80 °C for later use. For the co-immunoprecipitation (CoIP) experiments, Pierce™ Co-Immunoprecipitation Kit (Thermo Fisher) was used according to the manufacturer’s instructions. Antibody targeting Flag, PPL, or MIF (details are listed in Supplementary Table S1) was coupled to protein A/G magnetic beads, and then the magnetic bead-antibody complexes were mixed and incubated with the cell lysates to capture the target proteins and their interacting proteins. After incubation, the magnetic beads were thoroughly washed with the lysis buffer to remove non-specifically bound proteins. Finally, the protein complexes bound to the magnetic beads were eluted using Pierce™ IgG Elution Buffer (pH 2.0)(Thermo Fisher).

For the CoIP/MS experiments, the elution products were submitted to Luming Bio (Shanghai) for sample pre-processing steps such as protein concentration determination and tryptic digestion. The immunoprecipitated protein complexes were then identified using liquid chromatography-tandem mass spectrometry (LC-MS/MS) technology to comprehensively analyze the interacting protein molecules of the target proteins. To improve specificity, proteins detected in the IgG control samples were excluded from further analysis. The remaining proteins identified in the Flag-DEFB1 pulldown samples were considered candidate interactors and were ranked according to their relative MS abundance values in each replicate dataset. Functional annotation was subsequently performed to identify proteins localized to the cell membrane or extracellular space.

For the Western blot experiments, the elution products were analyzed by Western blot to verify the interactions between specific proteins. The secondary antibody used was VeriBlot for IP Detection (HRP) (Abcam, #ab131366, 1:1000), which only recognizes the native form of the primary antibody and thus does not detect the denatured heavy and/or light chains of the primary antibody during Western blot analysis [25].

### Macrophage polarization

Phorbol 12-myristate 13-acetate (PMA) (100 μg/mL, Sigma-Aldrich, St. Louis, MO, USA) was added to prime the THP-1 monocytes into macrophage-like cells (M0) for 48 h. M0 cells were further differentiated into M1 cells with exposure to 20 ng/mL interferon γ (IFN-γ) (Proteintech, Wuhan, Hubei, China) and 100 ng/mL lipopolysaccharide (LPS) (Proteintech), and into M2 cells with exposure to 20 ng/mL interleukin (IL)-4 (Proteintech) and IL-13 (Proteintech) for another 48 h.

### Reagent

ISO-1 (#T3680) was obtained from TargetMol (Boston, Massachusetts, USA).

### Antibody construction

The DEFB1-blocking monoclonal antibody was custom-made by Abclonal (Wuhan, Hubei, China). The brief procedure is outlined below:

First, a DEFB1 template plasmid was prepared and underwent in vitro transcription. The resulting mRNA was encapsulated within lipid nanoparticles and used as an antigen to immunize five rabbits. After three rounds of immunization, serum titers were measured. Once the titer reached 1:50,000, two boost immunizations were administered. The rabbit with the highest final titer was then selected. B cells were then isolated from the spleen using flow cytometry. The genes encoding the antibody heavy and light chains were amplified by PCR and randomly recombined. The recombinant single-chain antibody genes were inserted into phages to construct a phage display library.

Next, DEFB1 was covalently immobilized on magnetic beads. The constructed phage display library was subjected to five rounds of affinity selection against the immobilized DEFB1 antigen. Finally, the six clones with the highest affinities were selected. Their DNA was extracted and sequenced. Based on the obtained sequences, plasmid vectors were constructed, transfected for expression, and the resulting antibodies were purified.

### Flow cytometry

To quantify the expression levels of CD68, CD86, and CD206, cells were harvested and incubated with corresponding antibodies (antibody details are provided in Supplementary Table 1) for 1 h on ice. The distributions of positive cells were then detected using the FACSAria III flow cytometry (BD, Franklin Lakes, NJ, USA). The acquired data were processed and visualized using FlowJo Software (version 10) (TreeStar, Woodburn, OR, USA).

### Patient-derived organoids (PDO)

Fresh tumor tissues resected from lung adenocarcinoma patients pathologically diagnosed at Zhongshan Hospital, Fudan University, were collected, minced, washed with cold buffer, and enzymatically digested. using an organoid digestive solution (D1Med, Hangzhou, China) as previously reported [26–28]. The digested products were centrifuged, and the precipitates were mixed with Matrigel (Absin, Shanghai, China) at a ratio of 25:1. The mixture was seeded into 24-well plates and allowed to solidify at 37 °C for 10 min. Subsequently, 500 μL of PDO medium (D1Med) was added to each well to generate organoids. For the DEFB1-blocking antibody experiment, the organoids were treated with a final concentration of 1 μg/mL of the DEFB1-blocking antibody mAb-5 or rabbit IgG for 7 days, with the medium changed every three days. The organoids were photographed under a microscope, and their viability was measured using the CellTiter-Lumi Luminometric 3D Cell Viability Assay Kit (Beyotime).

### Tumor xenografts

All animal experiments were strictly conducted in accordance with the relevant regulations of the Animal Ethics Committee of Zhongshan Hospital, Fudan University. Four-week-old male BALB/c nude mice were purchased from Gempharmatech (Shanghai, China) and housed under pathogen-free conditions. Cells (1 × 10^6^) were suspended in 100 μL of cold PBS and subcutaneously injected into the right flanks of each nude mouse. The tumor volume was measured every three days using a caliper until the end of the experiment and calculated according to the formula *v* = length × width^2^ × 1/2. For the DEFB1 monoclonal antibody treatment experiment, when the tumor volume reached 200 mm^3^, the mice were intraperitoneally injected with the DEFB1 monoclonal antibody mAb-5 or control IgG at a dose of 60 μg per injection, every 3 days, for a total of 9 times. At the end of the experiment, the transplanted tumors were weighed, and the tumor tissues were collected for subsequent multiplex immunohistochemical detection.

### Spontaneous lung cancer model

Both C57BL/6 mice with humanized DEFB1 and C57BL/6 mice carrying a Cre-LoxP-mediated Kras G12D mutation were obtained from Shanghai Model Organisms Center. These mice were bred to generate eight-week-old male C57BL/6 mice that were both humanized for DEFB1 and carried the Cre-LoxP-mediated Kras G12D mutation. Then, to induce lung adenocarcinoma, 5 × 10^10^ copies of Scgb1a1-Cre adeno-associated virus (AAV) (GeneChem) were intranasally instilled to remove the stop element and activate the expression of oncogenic Kras as previously reported [29]. Eight weeks after AAV instillation, the tumor formation in the lungs of the mice was detected using chest CT (Quantum GX2 Micro-CT Scanner). In the following 4 weeks, the mice were administered intraperitoneal injections of either the DEFB1 monoclonal antibody mAb-5 or control IgG at a dose of 60 μg per injection, every 3 days, for a total of 9 times. Subsequently, the tumor status in the lungs was assessed using chest CT.

### Safety evaluation of mAb-5 in healthy immunocompetent mice

To assess the safety profile of mAb-5, eight-week-old healthy male C57BL/6 mice with humanized DEFB1 (Shanghai Model Organisms Center) were intraperitoneally injected with the DEFB1 monoclonal antibody mAb-5 or control IgG at a dose of 60 μg per injection, every 3 days, for a total of 9 times. Throughout the study, mice were monitored daily for behavioral changes, including low activity, shaking, and nodding respiration. Body weight was measured weekly. At the study endpoint (Day 28), blood samples were collected via cardiac puncture for blood biochemistry analysis by Shanghai Model Organisms Center. Major organs (heart, lungs, liver, spleen, and kidneys) were harvested, fixed in 4% paraformaldehyde, embedded in paraffin, and sectioned for hematoxylin and eosin (HE) staining.

### Statistical analysis

All experiments were performed independently and repeated at least three times to ensure the reliability of the results. Continuous variables between two or multiple groups were compared using unpaired Student’s *t*-test, one-way analysis of variance (ANOVA), or two-way ANOVA, respectively, while Bonferroni correction was used as a correction method for multiple comparisons among groups. The Mann–Whitney *U*-test was used to compare groups defined by categorical variables. Survival analysis was performed using the Kaplan–Meier (KM) and Log-rank methods. Unless otherwise specified, the results are presented as the mean value, and the error bars represent the standard deviation. All statistical analyses were performed using GraphPad Prism software (version 9.0) and R software. *p*-values were two-sided, and *p* < 0.05 was considered statistically significant.

## Results

### DEFB1 exhibits elevated expression and indicates unfavorable survival in lung adenocarcinoma

**Fig. 1:**
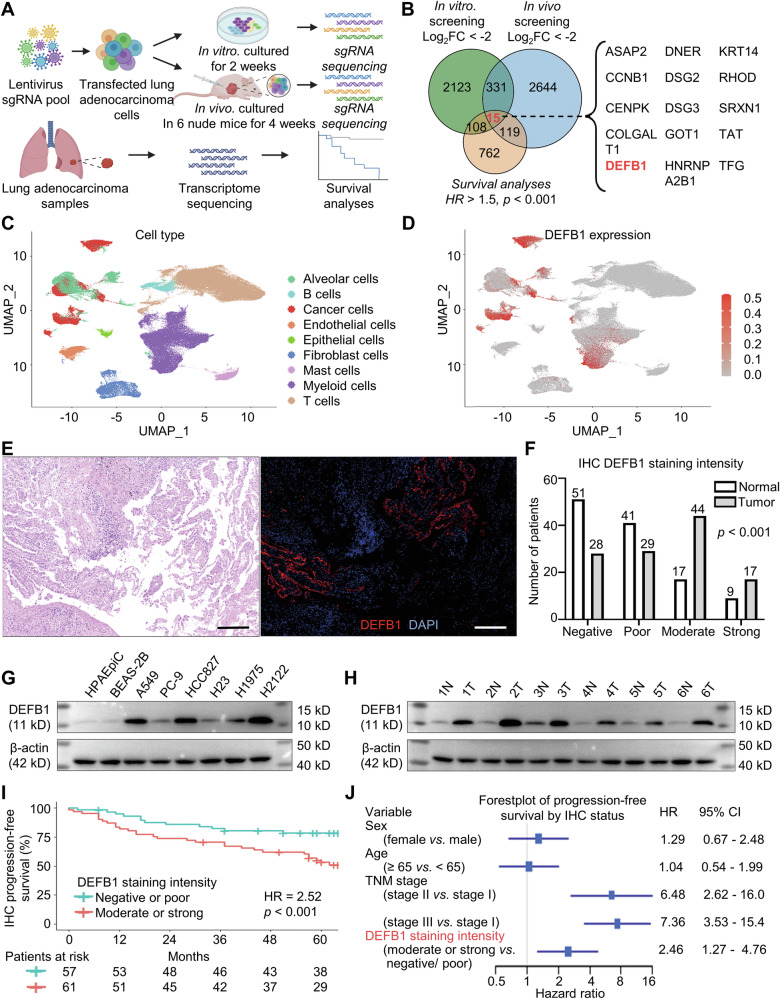
DEFB1 exhibits elevated expression and indicates unfavorable survival in lung adenocarcinoma. **A**, **B** Intersection of genes that might promote lung adenocarcinoma proliferation obtained from in vitro and in vivo CRISPR/Cas9 genome-wide knockout experiments and genes associated with worse prognosis of lung adenocarcinoma; **C**, **D** UMAP clustering of single-cell transcriptome sequencing data for cell clustering and DEFB1 expression levels in each single cell; **E**, **F** DEFB1 expression in 118 pairs of lung adenocarcinoma tumor tissues and adjacent normal lung tissues by multiple immunohistochemical staining (red) (scale bar: 200 μm); **G**, **H** Western blot results of DEFB1 expression in normal lung epithelial cell lines, lung adenocarcinoma cell lines, as well as six pairs of tumor tissues and adjacent normal tissues obtained from patients with lung adenocarcinoma; **I**, **J** Kaplan–Meier and Cox survival analyses between tumor DEFB1 expression and progression-free survival in lung adenocarcinoma patients based on multiple immunohistochemical staining results. Statistical analysis: **F** Mann–Whitney *U*-test, and **G** Log-rank test.

This study aimed to identify genes crucial for lung adenocarcinoma proliferation through a combination of in vitro and in vivo whole-genome knockout experiments and survival analysis. By screening genes that might promote lung adenocarcinoma proliferation in the whole-genome knockout experiments (Fig. 1A, Supplementary Tables 2 and 3) and identifying those whose expression was significantly correlated with worse patient prognosis (Fig. 1A, Supplementary Table 4) using the data of 496 lung adenocarcinoma samples in the TCGA database, we intersected the candidate genes from both approaches and identified 15 candidate genes (Fig. 1B). Among these, DEFB1, whose role in cancers remains largely unexplored, was selected for further investigation due to its potential functions and novelty.

Further analysis of our previous high-throughput single-cell sequencing data of lung adenocarcinoma revealed distinct expression patterns of DEFB1 [30]. The data, encompassing 204,157 cells from 12 normal lung tissue samples, 11 early-stage lung adenocarcinoma samples, and 6 advanced-stage lung adenocarcinoma samples, showed that DEFB1 was significantly overexpressed in lung adenocarcinoma cells and had some expression in myeloid cells, while being nearly absent in T cells, B cells, fibroblasts, and macrophages (Fig. 1C, D).

To validate the differential expression of DEFB1 between lung adenocarcinoma cells and normal cells, multiplex immunohistochemical staining was performed to detect DEFB1 expression in 118 pairs of surgical resection tissues from lung adenocarcinoma patients treated in our institution. The results consistently confirmed that DEFB1 expression was significantly higher in lung adenocarcinoma cells than in normal cells (Fig. 1E, F). Additionally, Western blot, qPCR, and ELISA experiments further supported these findings, showing markedly elevated expression and secretion of DEFB1 in a panel of lung adenocarcinoma cell lines (A549, PC-9, HCC827, H23, H1975, and H2122) are markedly elevated compared to those in normal lung epithelial cell lines (HPAEpiC and BEAS-2B) (Fig. 1G, Supplementary Fig. 1A, B). Western blot analysis of tissues from six lung adenocarcinoma patients also indicated significantly higher DEFB1 expression in tumor tissues compared to adjacent normal tissues (Fig. 1H).

Survival analysis based on our multiplex immunohistochemical staining results indicated that patients with high DEFB1 expression in tumor cells had both a worse progression-free survival (HR = 2.52, *p* < 0.001) (Fig. 1I) and overall survival prognosis (HR = 2.26, *p* = 0.039) (Supplementary Fig. 1C). The forests plots from the multivariate Cox analysis based on immunohistochemical results also suggested that DEFB1 expression was an independent predictor of patient prognosis (Fig. 1J, and Supplementary Fig. 1D).

Furthermore, analyses of the GEO datasets integrated on the Kmplot website and the TCGA dataset both demonstrated a significant correlation between high DEFB1 expression and poorer patient prognosis, consistent with the immunohistochemical findings (Supplementary Fig. 1E, F). The forest plots of the multivariate Cox analyses based on the TCGA data further emphasized DEFB1 expression as an independent unfavorable predictor of patient prognosis (Supplementary Fig. 1G). Meanwhile, analysis of TCGA data across several tumor types beyond lung adenocarcinoma, such as lung squamous cell carcinoma, kidney renal clear cell carcinoma, and thyroid carcinoma, has revealed a significant upregulation of DEFB1 expression compared to their respective normal tissue counterparts, a finding that may warrant further investigation (Supplementary Fig. 1H–J).

### DEFB1 enhances lung adenocarcinoma proliferation, migration, and invasion in vitro and tumorigenesis in vivo

**Fig. 2:**
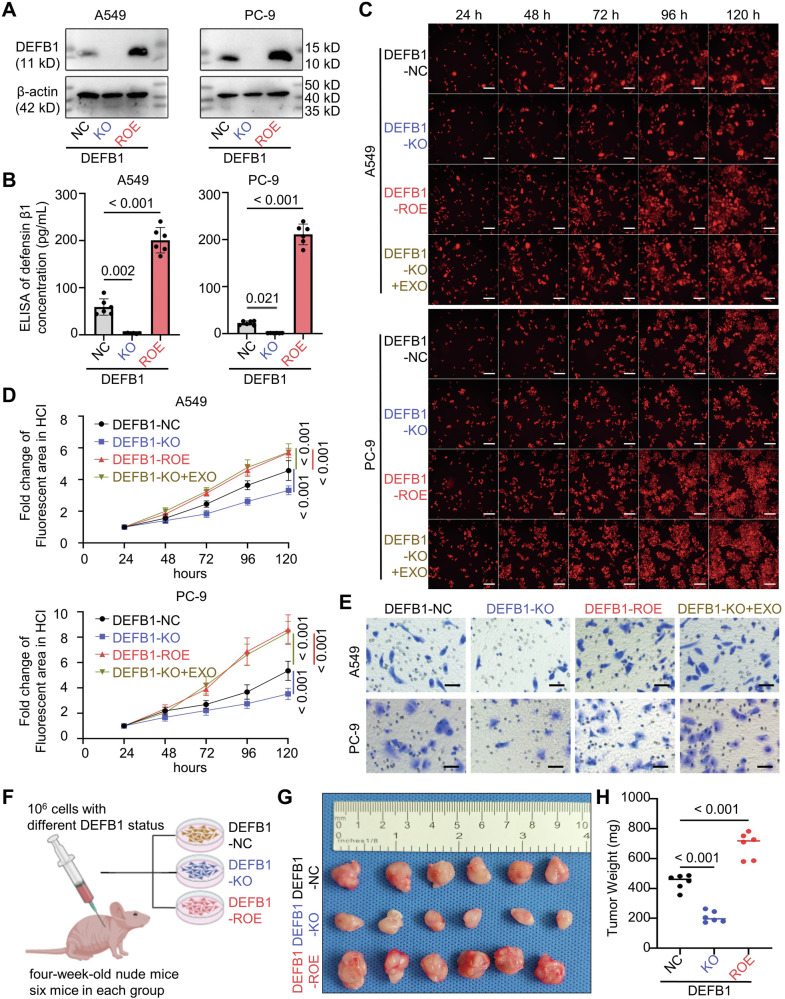
DEFB1 enhances lung adenocarcinoma proliferation, migration, and invasion in vitro and tumorigenesis in vivo. **A**, **B** Western blot and ELISA for DEFB1 protein expression in A549/PC-9 DEFB1-NC/KO/ROE cells; **C**, **D** High-content imaging (HCI) for the proliferation of A549/PC-9 DEFB1-NC/KO/ROE/KO + EXO cells (scale bar: 200 μm); **E** Transwell assay for the invasion capacity of A549/PC-9 DEFB1-NC/KO/ROE/KO + EXO cells (scale bar: 100 μm); **F** Schematic diagram of for the xenograft experimental process; **G**, **H** In vivo proliferation and final tumor weights of A549 DEFB1-NC/KO/ROE cells. Statistical analysis: **B**, **H** One-way ANOVA and Bonferroni correction; **D** Two-way ANOVA test (*n* = 6) and Bonferroni correction.

To validate the impact of DEFB1 on lung adenocarcinoma cells in vitro, this study employed CRISPR/Cas9 technology to knock out (KO) DEFB1 in two of the most frequently utilized lung adenocarcinoma cell lines, A549 and PC-9, which harbor KRAS and EGFR mutations, respectively, representing the predominant driver gene mutations observed in lung adenocarcinoma. The knockout efficiency was verified using Western blot and ELISA (Fig. 2A, B). High-content imaging (HCI) and Alamar blue assay both confirmed that DEFB1-KO significantly inhibited the proliferation of lung adenocarcinoma cells (Fig. 2C, D, Supplementary Fig. 2A).

To confirm that the observed changes in proliferation capacity were specifically attributable to DEFB1 knockout rather than off-target effects, we re-overexpressed DEFB1 with mutated sgRNA-targeted sites (ROE) in A549/PC-9 DEFB1-KO cells, and our Western blot and ELISA confirmed the re-overexpressed DEFB1 protein in cells and culture supernatant (Fig. 2A, B). Our HCI and Alamar blue assay both demonstrated that DEFB1-ROE significantly rescued the proliferation inhibition caused by DEFB1-KO (Fig. 2C, D, Supplementary Fig. 2A).

Meanwhile, our results in Fig. 2B indicate that there seems to be an upper limit of approximately 200 pg/mL for the DEFB1 protein concentration in the culture supernatant. Therefore, considering that DEFB1 is a secreted protein, we added exogenous DEFB1 (200 pg/mL) to the culture media of DEFB1-KO cells (KO + EXO). The DEFB1-KO + EXO also significantly rescued the proliferation inhibition induced by DEFB1-KO (Fig. 2C, D, Supplementary Fig. 2A). These findings collectively validate that DEFB1 promotes the proliferation of lung adenocarcinoma cells in vitro.

Transwell assays provided further evidence that DEFB1-KO markedly impaired the migratory and invasive capacities of lung adenocarcinoma cells (Fig. 2E, Supplementary Fig. 2B, C). Notably, either DEFB1-ROE or DEFB1-KO + EXO was able to counteract the suppression of migration and invasion induced by DEFB1-KO, also shown in Fig. 2E and Supplementary Fig. 2B, C.

Furthermore, in vivo cell-derived xenograft conducted in nude mice demonstrated that DEFB1-KO diminished the in vivo proliferation potential of A549 cells, whereas DEFB1-ROE markedly augmented the in vivo proliferation capacity (Fig. 2F–H).

### DEFB1 interacts with Periplakin (PPL) to promote epithelial-to-mesenchymal transition (EMT) and proliferation in lung adenocarcinoma cells

**Fig. 3:**
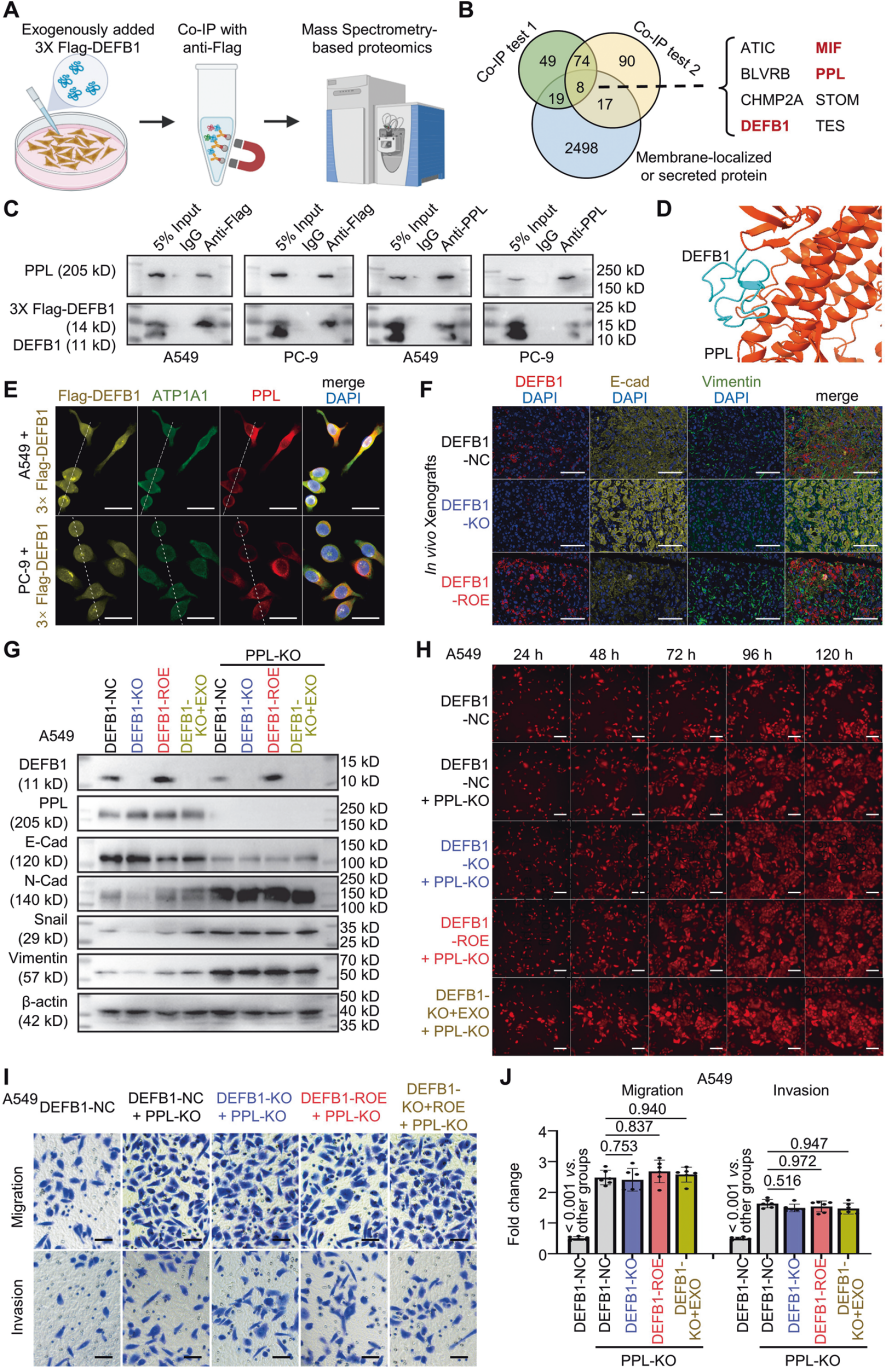
DEFB1 interacts with Periplakin (PPL) to promote epithelial-to-mesenchymal transition (EMT) and proliferation in lung adenocarcinoma cells. **A**, **B** Flowchart and results for screening interacting membrane-localized and secreted proteins using co-immunoprecipitation (CoIP) combined with mass spectrometry and anti-Flag in A549 cells treated with exogenously added 3× Flag-tagged DEFB1 (200 pg/mL); **C** CoIP verification of the interaction between DEFB1 and PPL in A549/PC-9 cells treated with exogenously added 3× Flag-tagged DEFB1 (200 pg/mL); **D** AlphaFold 3 prediction of the interaction mode between DEFB1 and PPL; **E** Immunofluorescence assay for detecting the cellular distribution of Flag-DEFB1, ATP1A1 (plasma membrane marker), and PPL in A549/PC-9 cells treated with exogenously added 3× Flag-tagged DEFB1 (200 pg/mL)(scale bar: 30 μm); White dashed lines indicate the regions used for fluorescence intensity profile analysis; **F** Epithelial-mesenchymal transition (EMT) status of nude-mouse xenografts derived from A549 DEFB1-NC/KO/ROE cells (scale bar: 100 μm); **G** Western blot detection of the expression of EMT-related proteins in A549 DEFB1-NC/KO/ROE/KO + EXO cells with or without PPL knockout (PPL-KO); **H** High-content imaging (HCI) for the proliferation of A549 DEFB1-NC, and DEFB1-NC/KO/ROE/KO + EXO cells with PPL-KO (scale bar: 200 μm); **I**, **J** Transwell assays of the migration and invasion capacities of A549 DEFB1-NC, and DEFB1-NC/KO/ROE/KO + EXO cells with PPL-KO (scale bar: 100 μm). Statistical analysis: **J** One-way ANOVA test and Bonferroni correction.

Given that DEFB1 is a secreted protein, and our results indicate that DEFB1-KO + EXO can effectively restore the tumor-suppressive effect in DEFB1-KO cells, we tried to explore its downstream mechanisms by adding exogenous 3× Flag-tagged DEFB1 into the growth media of A549 cells in culture, and employing Flag-antibody-based CoIP/MS to identify its interacting proteins (Supplementary Table 5), with a particular focus on those localized to the cell membrane and extracellular space (Fig. 3A, B).

After excluding proteins detected in the IgG control samples, the remaining proteins were ranked according to their MS abundance values in each dataset. Under these criteria, DEFB1 ranked 17th and 13th in the two replicate datasets, respectively, supporting the robustness and reproducibility of the CoIP/MS analysis (Supplementary Table 5). Functional annotation was subsequently performed to identify membrane and extracellular proteins for further analysis (Fig. 3A, B, Supplementary Table 6).

In addition to DEFB1 itself, seven other candidate genes were identified at the intersection (Fig. 3B). Among them, Periplakin (PPL), a cell-membrane-localized component of intercellular desmosomal junctions reported to potently inhibit epithelial-mesenchymal transition (EMT) and cell proliferation, caught our attention [31, 32].

To validate the potential interaction between DEFB1 and PPL, we added exogenous 3× Flag-tagged DEFB1 into the growth media of A549 and PC-9 cells in culture, followed by CoIP with Flag and PPL antibodies. Western blot results clearly demonstrated a robust interaction between DEFB1 and PPL (Fig. 3C). Furthermore, predictions based on AlphaFold 3.0 provide insights into the binding mode of DEFB1 with PPL (Fig. 3D) [33, 34].

Meanwhile, we conducted RNA-Seq analysis to identify the differentially expressed genes between A549/PC-9 DEFB1-KO + EXO cells and DEFB1-KO cells. Notably, the results indicated a significant enrichment of these differentially expressed genes in EMT-related pathways (Supplementary Fig. 3A, B), suggesting that PPL/EMT may serve as the downstream axis of DEFB1.

Furthermore, exogenous 3× Flag-tagged DEFB1 was added to the culture media of A549 and PC-9 cells, and the cellular distribution of Flag-DEFB1, the plasma membrane marker ATP1A1, and PPL was examined by immunofluorescence staining (Fig. 3E). Due to the lack of suitable antibodies for direct immunocytochemical detection of DEFB1, Flag-tagged DEFB1 was used as a surrogate. Flag-DEFB1 displayed a peripheral staining pattern and showed strong spatial overlap with the membrane marker ATP1A1, and line-scan fluorescence intensity analysis further demonstrated a high degree of correlation between Flag-DEFB1 and ATP1A1, supporting its membrane association (Fig. 3E, Supplementary Fig. 3C, D). In contrast, PPL showed a predominantly membrane and cytoplasmic distribution with reduced nuclear localization (Fig. 3E). These observations are consistent with previous reports that DEFB1 can associate with cellular membranes, likely due to its high content of positively charged amino acids, and provide supportive evidence for a potential interaction between DEFB1 and PPL, in line with the CoIP/MS results [35].

In addition, based on the results of multiplex immunohistochemical staining of nude-mouse xenografts listed in Fig. 2G, it was demonstrated that in vivo xenografts of A549 DEFB1-KO cells exhibited higher E-cadherin (E-cad) expression and lower Vimentin expression (Fig. 3F). Conversely, the opposite trend was observed in the xenografts of A549 DEFB1-ROE cells (Fig. 3F). Meanwhile, Western blot analysis in A549/PC-9 DEFB1-NC/KO/ROE/KO + EXO cells, revealed that knocking out DEFB1 significantly altered the expression levels of a series of EMT-related proteins. Specifically, it promoted the expression of E-cad while inhibiting the expression of N-cadherin (N-Cad), Vimentin, and Snail (Fig. 3G, Supplementary Fig. 3E). Notably, the effects of DEFB1 knockout on EMT-related protein expression were completely reversed in DEFB1-ROE/KO + EXO cells (Fig. 3G, Supplementary Fig. 3E). These findings collectively suggest that DEFB1 significantly promotes the EMT process.

To verify that PPL acts as the key mediator in DEFB1’s effect on EMT, we knocked out PPL in A549/PC-9 DEFB1-NC/KO/ROE/KO + EXO cells. Western blot analysis revealed that PPL knockout not only significantly promoted EMT but also substantially eliminated the variations in the expression levels of EMT-related proteins across the NC, KO, ROE, and KO + EXO cell groups (Fig. 3G, Supplementary Fig. 3E).

Furthermore, both our high-content analysis and Alamar blue assay demonstrated that knocking out PPL could significantly block the effect of DEFB1 on cell proliferation (Fig. 3H, Supplementary Fig. 3F–J). Meanwhile, our transwell experiments confirmed that knocking out PPL could markedly inhibit the impact of DEFB1 on cell migration and invasion (Fig. 3I, J, Supplementary Fig. 3K, L).

In summary, our findings strongly suggest that DEFB1 interacts with PPL, thereby influencing epithelial-mesenchymal transition (EMT), proliferation, migration, and invasion in lung adenocarcinoma.

### DEFB1 interacts with macrophage Migration Inhibitory Factor (MIF) to promote M2 polarization of macrophages in vitro and in vivo

**Fig. 4:**
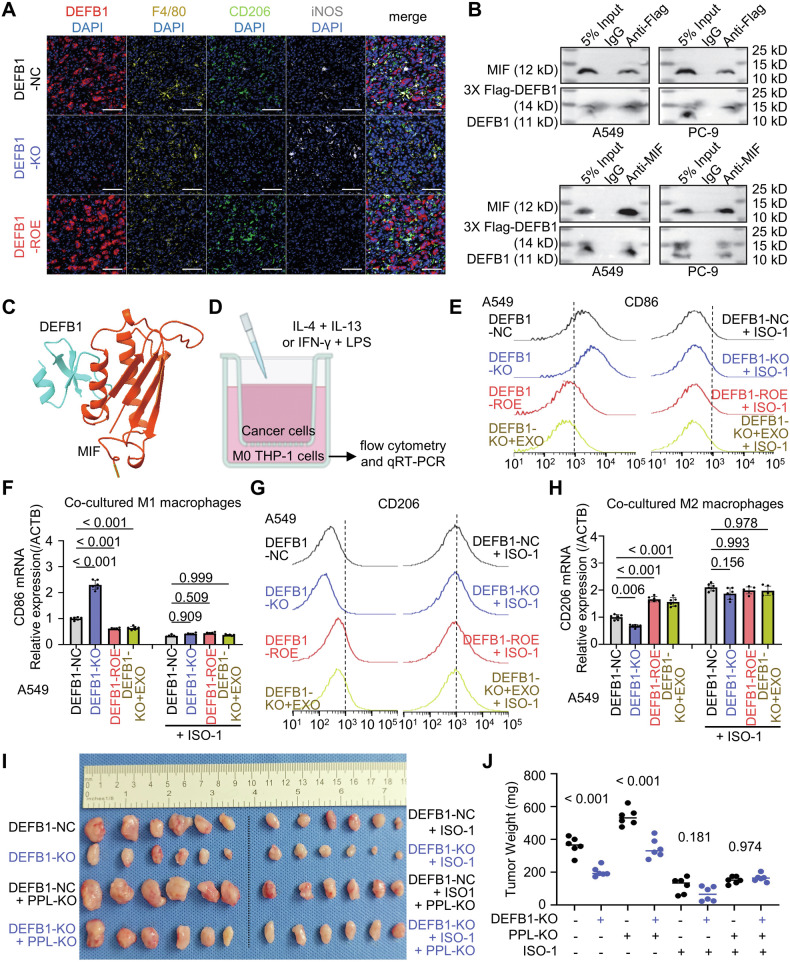
DEFB1 interacts with macrophage Migration Inhibitory Factor (MIF) to promote M2 polarization of macrophages in vitro and in vivo. **A** Infiltrating status of M1 (marked by F4/80 and INOS) and M2 (marked by F4/80 and CD206) macrophages in nude-mouse xenografts derived from A549 DEFB1-NC/KO/ROE cells (scale bar: 200 μm); **B** CoIP verification of the interaction between DEFB1 and MIF in A549/PC-9 cells treated with exogenously added 3× Flag-tagged DEFB1 (200 pg/mL); **C** AlphaFold 3 prediction of the interaction mode between DEFB1 and MIF; **D** Schematic diagram of an in vitro co-culture model of lung adenocarcinoma cells and THP-1 derived macrophages; **E**, **F** Flow Cytometry and qRT-PCR for evaluating the effects of DEFB1 and/or MIF inhibitor (ISO-1) on M1 polarization of THP-1 M0 cells co-cultured with A549 cells; **G**, **H** Flow Cytometry and qRT-PCR for evaluating the effects of DEFB1 and/or ISO-1 on M2 polarization of THP-1 M0 cells co-cultured with A549 cells; **I**, **J** Effects of DEFB1-KO, PPL-KO, and/or ISO-1 on the in vivo proliferative capacity in nude-mouse CDX model. Statistical analysis: (**F**, **H**, and **G**) One-way ANOVA test and Bonferroni correction.

After carefully analyzing the DEFB1 CoIP/MS results shown in Fig. 3B, we observed that DEFB1 also interacts with MIF. Notably, the full name of the MIF gene underscores its significant impact on macrophages, and several previous studies have reported that MIF significantly promotes M1 polarization while inhibiting M2 polarization of macrophages in cancers, suggesting its potential as a therapeutic target for tumor treatment, while DEFB1 is also reported to be associated with macrophage infiltration in lung adenocarcinoma [36–39]. Furthermore, we examined the infiltration of M1 and M2 macrophages in nude-mouse xenograft samples derived from A549 DEFB1-NC/KO/ROE cells, as mentioned in Fig. 2G. The results demonstrated that DEFB1 significantly reduced M1 polarization (marked by F4/80 and INOS) of infiltrating macrophages in nude-mouse tumor samples, while promoting M2 polarization (marked by F4/80 and CD206) (Fig. 4A). Consequently, we hypothesized that DEFB1 might influence macrophage polarization through its interaction with MIF.

To validate the binding interaction between DEFB1 and MIF, we performed subsequent CoIP experiments, employing Flag and MIF antibodies in combination with Western blot analysis. These experiments provided comprehensive confirmation of the binding between DEFB1 and MIF (Fig. 4B). Additionally, predictions from AlphaFold 3 elucidated the binding mode of DEFB1 with MIF (Fig. 4C).

In vitro co-culture experiments involving A549/PC-9 DEFB1-NC/KO/ROE/KO + EXO cells, alongside the THP-1 monocytic cell line, demonstrated that DEFB1 significantly inhibited the polarization of M0 macrophages towards the M1 phenotype while concurrently promoting their polarization to the M2 phenotype (Fig. 4D–H, Supplementary Fig. 4A–D). Moreover, the addition of the MIF inhibitor ISO-1 in the in vitro co-culture experiments effectively attenuated the DEFB1-induced polarization shift (Fig. 4D–H, Supplementary Fig. 4A–D). Collectively, these results comprehensively underscore the pivotal role of DEFB1 in promoting M2 macrophage polarization through its interaction with MIF.

In vivo experiments employing nude-mouse xenograft models demonstrated that either the knockout of PPL or the administration of the MIF inhibitor ISO-1 partially blocked the tumor-promoting effects of DEFB1 (Fig. 4I, J). However, the combination of PPL knockout and ISO-1 treatment effectively abolished the tumor-enhancing activity of DEFB1, conclusively underscoring the critical involvement of both PPL and MIF in mediating the in vivo tumorigenic effects of DEFB1 (Fig. 4I, J).

### Anti-DEFB1 monoclonal antibody as a promising therapeutic agent against lung adenocarcinoma

A compelling therapeutic strategy involves utilizing specific monoclonal antibodies to precisely target secreted proteins and neutralize their activities, thereby conferring therapeutic advantages across various diseases, as exemplified by bevacizumab targeting VEGF and adalimumab targeting TNF-α [40–43]. This raises the intriguing possibility of utilizing specific monoclonal antibodies to block DEFB1, a secreted protein, as a therapeutic modality for lung adenocarcinoma.

**Fig. 5:**
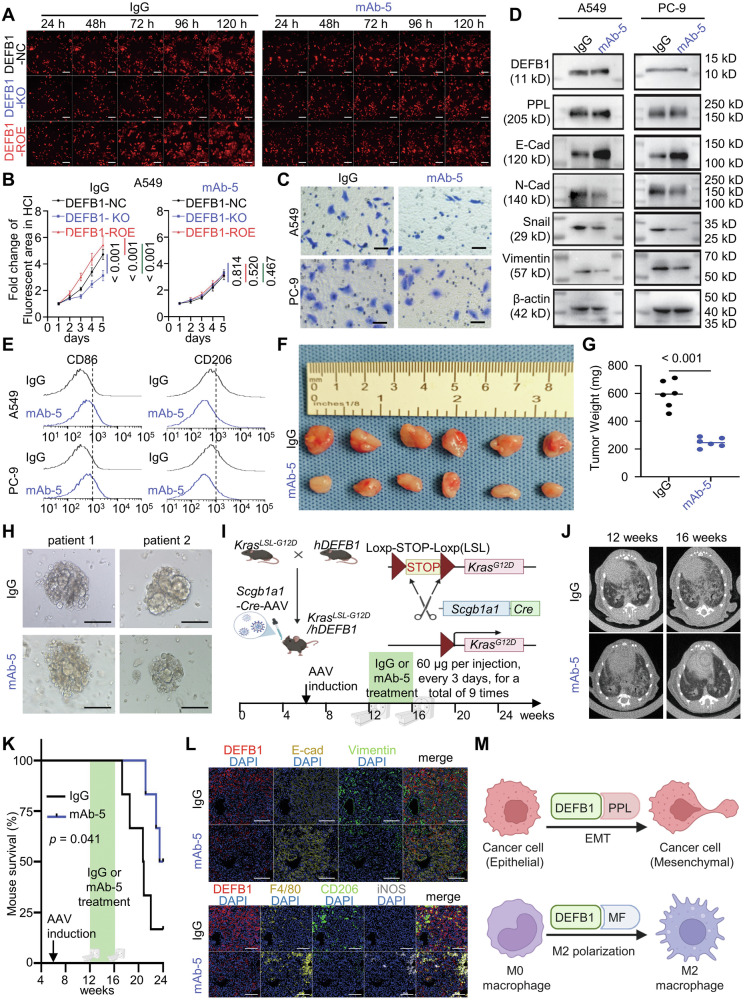
Anti-DEFB1 monoclonal antibody as a promising therapeutic agent against lung adenocarcinoma. **A**, **B** High-content imaging for the proliferation of A549 DEFB1-NC/KO/ROE cells treated with 1 μg/mL IgG or anti-DEFB1 monoclonal antibody mAb-5 (scale bar: 200 μm); **C** Transwell assay for the invasion capacity of A549/PC-9 cells treated with 1 μg/mL IgG or mAb-5 (scale bar: 100 μm); **D** Western blot detection of the expression of epithelial-mesenchymal transition (EMT)-related proteins in A549/PC-9 cells treated with 1 μg/mL IgG or mAb-5; **E** Flow Cytometry for evaluating the M1/M2 polarization statues of THP-1 M0 cells co-cultured with A549/PC-9 cells treated with 1 μg/mL IgG or mAb-5; **F**–**H** In vivo proliferative capacity in nude mice CDX model and in human lung adenocarcinoma organoids (scale bar: 100 μm) treated with IgG or mAb-5 (60 μg per injection, every 3 days, for a total of 9 times); **I** Schematic description of *Kras*^*G12D*^-driven spontaneous lung tumor mice model expressing humanized DEFB1 and the treatment mode; **J**, **K** Representative images of chest computed tomography before and after the treatments of IgG or mAb-5 (60 μg per injection, every 3 days, for a total of 9 times) and survival analysis in spontaneous lung cancer model; **L** Epithelial-mesenchymal transition (EMT) and macrophage infiltrating statuses in spontaneous lung cancer model treated with IgG or mAb-5 (scale bar: 200 μm); **M** Mechanistic schema of DEFB1 in lung adenocarcinoma. Statistical analysis: **B** Two-way ANOVA test (*n* = 6) and Bonferroni correction; **G** Student’s *t*-test; **K** Log-rank test (*n* = 6).

To this end, we developed six monoclonal antibodies against human DEFB1. High-content imaging and Alama blue analyses revealed that mAb-5, compared to the other five antibodies and the control IgG, demonstrated superior efficacy in inhibiting the proliferation of A549 and PC-9 cells (Supplementary Fig. 5A, B). Further investigations conducted on cells with A549/PC-9 DEFB1-KO/ROE cells revealed that mAb-5 had no significant effect on the proliferation of DEFB1-KO cells, while it abolished the pro-proliferative effect induced by DEFB1 re-overexpression (Fig. 5A, B, Supplementary Fig. 5C–F), thus demonstrating that mAb-5 inhibits tumor proliferation by specifically suppressing DEFB1.

Transwell migration and invasion assays unequivocally demonstrated that mAb-5 significantly impeded the migratory and invasive capacities of A549 and PC-9 cells compared to the control IgG (Fig. 5C, Supplementary Fig. 5G, H). Detailed examination of EMT-associated proteins revealed that mAb-5 potently inhibited the epithelial-mesenchymal transition (EMT) process in lung adenocarcinoma cell lines (Fig. 5D). Furthermore, co-culture studies employing the THP-1 macrophage cell line and lung adenocarcinoma cell lines highlighted that mAb-5 significantly attenuated the polarization of M0 macrophages towards the M2 phenotype while promoting their polarization towards the M1 phenotype (Fig. 5E, Supplementary Fig. 5I).

The therapeutic potential of mAb-5 was further evaluated in both lung adenocarcinoma organoids and in vivo tumorigenesis models. Concurrently, results from nude-mouse xenograft experiments clearly demonstrated that mAb-5 significantly inhibited tumor growth in vivo and led to a reduction in the tumor weight (Fig. 5F, G). Our lung adenocarcinoma organoid models also validated the potent tumor proliferation-inhibitory effects of mAb-5 (Fig. 5H). Furthermore, we intranasally administered adeno-associated virus (AAV) carrying the Scgb1a1-Cre to specifically induce the expression of Kras^G12D^ in lung epithelial cells, thereby triggering the development of lung adenocarcinoma in C57BL/6 mice with humanized DEFB1 (Fig. 5I). In this spontaneous lung adenocarcinoma model with an intact immune system, we similarly observed that mAb-5 significantly retarded tumor growth and prolonged mouse survival (Fig. 5J, K).

Meanwhile, multiplex immunohistochemical analysis of both nude-mouse xenografts and spontaneous lung adenocarcinoma models revealed that mAb-5 significantly inhibits EMT in tumor cells and reduces M2 macrophage infiltration (Fig. 5L, Supplementary Fig. 5J, K). However, our results showed that mAb-5 does not significantly influence the infiltration capacity of CD8^+^ T cells (Supplementary Fig. 5L). This is likely attributable to the fact that KRAS-mutant lung cancers are characterized as immunologically “cold” tumors, which typically exhibit a lower number of infiltrating T cells [44, 45]. Collectively, these findings underscore the robust antitumor capabilities of mAb-5 across both organoid and in vivo. models.

To comprehensively evaluate the safety profile of mAb-5, we conducted a four-week treatment with mAb-5 or IgG in healthy immunocompetent C57BL/6 mice with humanized DEFB1. No discernible alterations in body weight, blood biochemistry, or behavioral patterns were noted in the mice. Additionally, hematoxylin and eosin (HE) staining of the vital organs, including the heart, lungs, liver, spleen, and kidney, failed to reveal any significant toxicological changes. Considering the high homology of about 90% between human and mouse PPL (88.2%) and MIF (89.6%) proteins, these mouse studies are deemed to reliably mimic human therapeutic responses, thereby indicating the potential safe application of mAb-5 in human therapy.

Collectively, our findings provide compelling evidence that DEFB1 functions as a novel driver in the pathogenesis of lung adenocarcinoma. Mechanistically, DEFB1 interacts with PPL to facilitate EMT, and it also engages with MIF to promote the M2 polarization of macrophages (Fig. 5M). Moreover, the anti-DEFB1 monoclonal antibody mAb-5 exhibits promising therapeutic potential.

## Discussion

This study systematically elucidated the pivotal role of DEFB1 in lung adenocarcinoma progression, identifying it as a novel driver of tumor advancement. Through a genome-wide CRISPR/Cas9 knockout screen combined with prognostic gene cross-analysis, we demonstrated that DEFB1 significantly promotes lung adenocarcinoma cell proliferation and invasion in vitro and in vivo, suggesting DEFB1 as a potential therapeutic target. High-throughput single-cell RNA-sequencing and immunohistochemistry revealed that DEFB1 is specifically overexpressed in lung adenocarcinoma cells compared to other cells within the tumor microenvironment, indicating a promising therapeutic specificity for DEFB1-targeted interventions.

In addition to our findings on DEFB1, our combined survival analysis of in vivo and in vitro genome-wide knockout screening results also identified 14 other genes of interest. Among them, DSG2, DSG3, and KRT14 have been reported as markers of lung squamous cell carcinoma [46, 47]. Our screening reveals that knocking out these genes markedly inhibits A549 cell proliferation, with their high expression in lung adenocarcinoma patients correlating to poor prognosis, possibly implicating involvement in the malignancy and drug-resistance-related transdifferentiation to lung squamous cell carcinoma [48]. CCNB1, CENPK, GOT1, HNRNPA2B1, RHOD, and SRXN1 have all been reported to be closely related to the proliferation and invasion of lung adenocarcinoma and may become key therapeutic targets for lung adenocarcinoma [49–54]. ASAP2, COLGALT1, DNER, TAT, and TFG have been less reported in lung cancer, and their roles in lung cancer warrant further in-depth investigation.

Human β-defensins, a family of epithelial cell-derived antimicrobial peptides, protect mucosal surfaces from microbial invasion [9, 10, 12]. Beyond their antimicrobial activity, β-defensins function as chemokines, regulating cell activation, proliferation, cytokine/chemokine production, migration, differentiation, angiogenesis, and wound healing [9, 10, 12]. Previous studies have reported that DEFB1 is upregulated in human lung squamous cell carcinoma and adenocarcinoma tissues compared to normal controls, with significantly elevated serum DEFB1 levels observed in lung cancer patients compared to healthy individuals [55, 56]. However, the role and mechanisms of DEFB1 in lung cancer remain poorly understood. In contrast to its tumor-suppressive effects in oral squamous cell carcinoma, head and neck cancer, and breast cancer [11, 57, 58], where DEFB1 reportedly inhibits tumor progression, the disparity may arise from distinct downstream signaling pathways in different tumor types, necessitating further investigation [13].

Our functional experiments further revealed that DEFB1 interacts with PPL to promote EMT and cell proliferation, while also interacting with MIF to induce macrophage polarization toward the M2 phenotype. EMT confers cancer cells with invasive and migratory capabilities, thereby serving as a core mechanism driving the malignant progression of lung adenocarcinoma and a critical therapeutic target [59–61]. Meanwhile, PPL functions as a cytolinker that bridges intermediate filaments with cytoskeletal proteins and is an essential component of desmosomal plaques, where it forms complexes with cadherins [31, 62]. Notably, PPL expression is frequently downregulated in various tumors, and its knockdown has been shown to enhance cellular proliferation, migration, invasion, and to confer an increased capacity for EMT initiation [32]. MIF has been identified as a multipotent cytokine secreted by various cell types involved in immune responses and physiological processes [37, 38]. For instance, MIF inhibits M1 polarization of macrophages, thereby positioning it as a potential therapeutic target in cancer [36, 63]. Collectively, these findings provide molecular evidence for DEFB1’s role in driving lung adenocarcinoma progression and modulating the immune microenvironment.

Antibody-based therapeutics have demonstrated remarkable success in oncology, particularly in targeting cell surface membrane proteins and secreted factors [64–66]. Their success has not only revolutionized cancer treatment paradigms but also spurred extensive research into the underlying mechanisms of tumor biology, driving the development of novel therapeutic strategies and paving the way for a new era of precision medicine in oncology. In this study, we customized a panel of anti-DEFB1 monoclonal antibodies and identified one of them, mAb-5, as the most promising candidate based on cellular assays. To further evaluate its therapeutic potential, we utilized organoid models, an emerging in vitro platform that recapitulates tumor heterogeneity and three-dimensional architecture, thereby faithfully reflecting tumor cell biology [67, 68]. The antitumor efficacy of the mAb-5 monoclonal antibody was also validated in mouse spontaneous lung cancer models, which closely mimic the tumorigenesis of human lung adenocarcinoma [69, 70]. Furthermore, its preclinical safety profile was initially assessed in mouse models. These findings not only substantiate DEFB1 as a viable therapeutic target but also provide robust preclinical evidence supporting its clinical translation.

Our study has several limitations. First, we cannot exclude the possibility that DEFB1 may also exert cytoplasmic functions. Such experimental restriction indeed introduces bias by focusing solely on membrane-localized or secreted proteins when using CoIP to identify DEFB1-interacting proteins. Second, our scRNA analysis reveals that DEFB1 shows high expression in both tumor cells and myeloid cells, strongly hinting at its potential to influence myeloid cell behavior and thus contribute to tumorigenesis. Nevertheless, our current study falls short of a comprehensive exploration of DEFB1’s specific effects on myeloid cells. We sincerely hope that future research will delve deeper into the interaction between DEFB1 and myeloid cells, thereby filling the existing knowledge gap and offering a fuller picture of DEFB1’s role in cancer. Third, several studies reported that DEFB1 weakens the function of CD8^+^ T cells [71]. Though our results revealed notably low CD8⁺ T cell infiltration in the mice's spontaneous lung tumors, this limited our ability to robustly compare the effects of mAb-5 on CD8⁺ T cell dynamics. But these findings from mouse models may not fully recapitulate the effects of mAb-5 in human lung cancer, given known interspecies differences in tumor immunobiology and the potential pleiotropic roles of DEFB1 in modulating the tumor microenvironment. Therefore, future studies are still warranted to dissect the immunoregulatory functions of DEFB1 in human lung cancer. Lastly, the current efficacy of mAb-5 in tumor suppression still has room for improvement. Future research could focus on optimizing the antibody sequence and design to enhance its therapeutic potential.

Collectively, our study identifies DEFB1 as a novel driver of lung adenocarcinoma, and the anti-DEFB1 monoclonal antibody mAb-5 emerges as a promising therapeutic candidate with significant potential.

## Supplementary information

Supplementary Figures

Supplementary Tables

Original Western blots

## Data Availability

The RNA-Seq data have been deposited in the Genome Sequence Archive, China National Center for Bioinformation (Project No.: PRJCA054106). The CoIP/MS data have been deposited in the OMIX, China National Center for Bioinformation (Project No.: OMIX013851). Other data generated or analyzed during this study are included in this published article and its supplementary information files.
