# Supplementary material for: Unveiling DEFB1 as a novel driver and promising therapeutic target in lung adenocarcinoma
Source: Cell Death Dis. 2026 Apr 20;17(1):519. doi: 10.1038/s41419-026-08748-4 (PMC13223247; doi:10.1038/s41419-026-08748-4)
Supplement: Supplementary file 1 — Supplementary Figures [file 41419_2026_8748_MOESM1_ESM.docx]

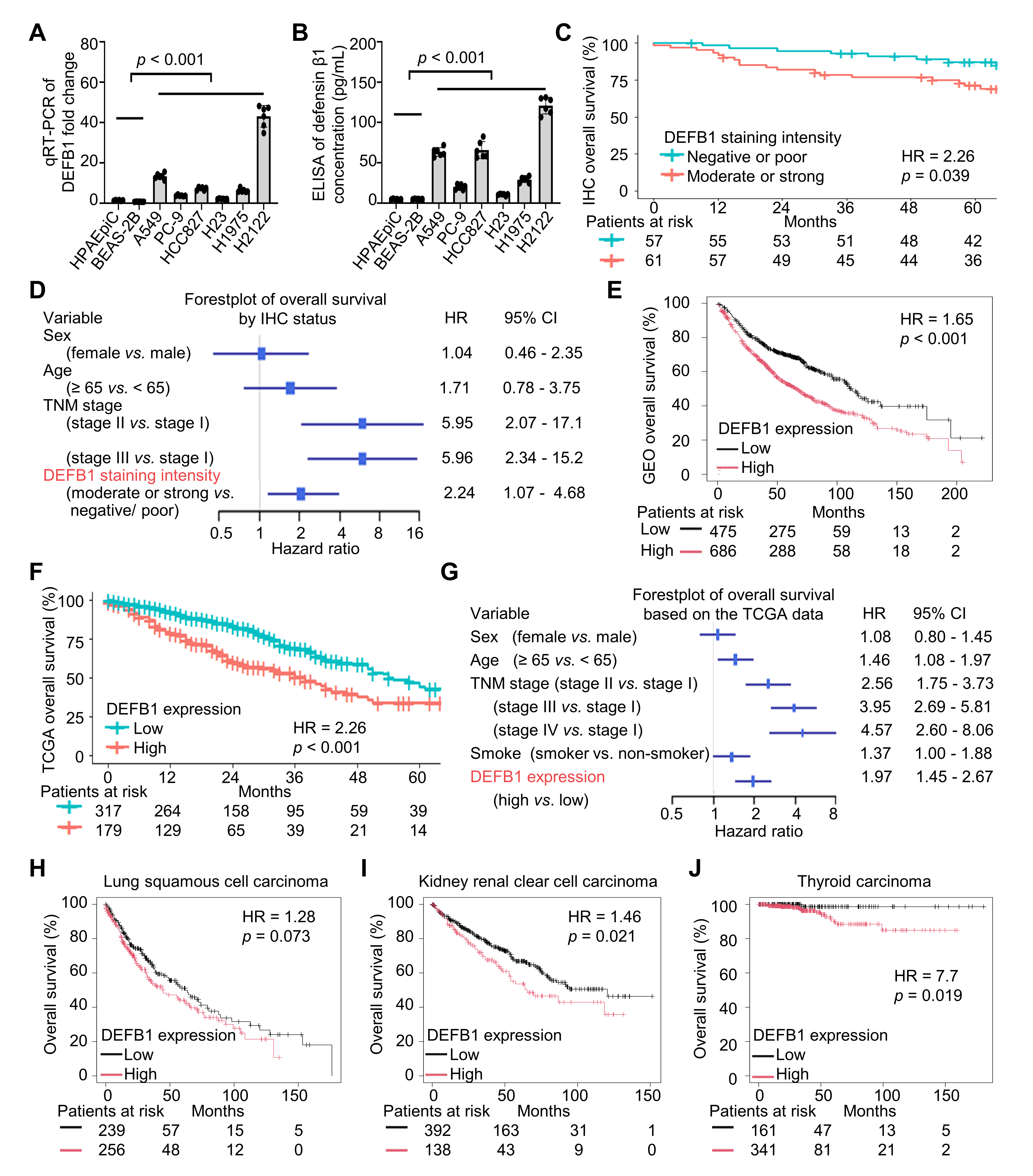


# Supplementary figure 1. DEFB1 exhibits elevated expression and indicates unfavorable survival in lung adenocarcinoma

(A-B) qRT-PCR and ELISA results of DEFB1 expression in normal lung epithelial cell lines and lung adenocarcinoma cell lines; (C-D) Kaplan-Meier and Cox survival analyses between tumor DEFB1 expression and overall survival in lung adenocarcinoma patients based on multiple immunohistochemical staining results; (E-G) Survival analyses between DEFB1 expression and overall survival in lung adenocarcinoma patients based on the GEO and the TCGA datasets integrated on the Kaplan-Meier plotter website; (H-J) Survival analyses between DEFB1 expression and overall survival in lung squamous cell carcinoma, kidney renal clear cell carcinoma, and thyroid carcinoma based on the GEO datasets integrated on the Kaplan-Meier plotter website. Statistical analysis: (E-F) and (H-J) Log-rank test.


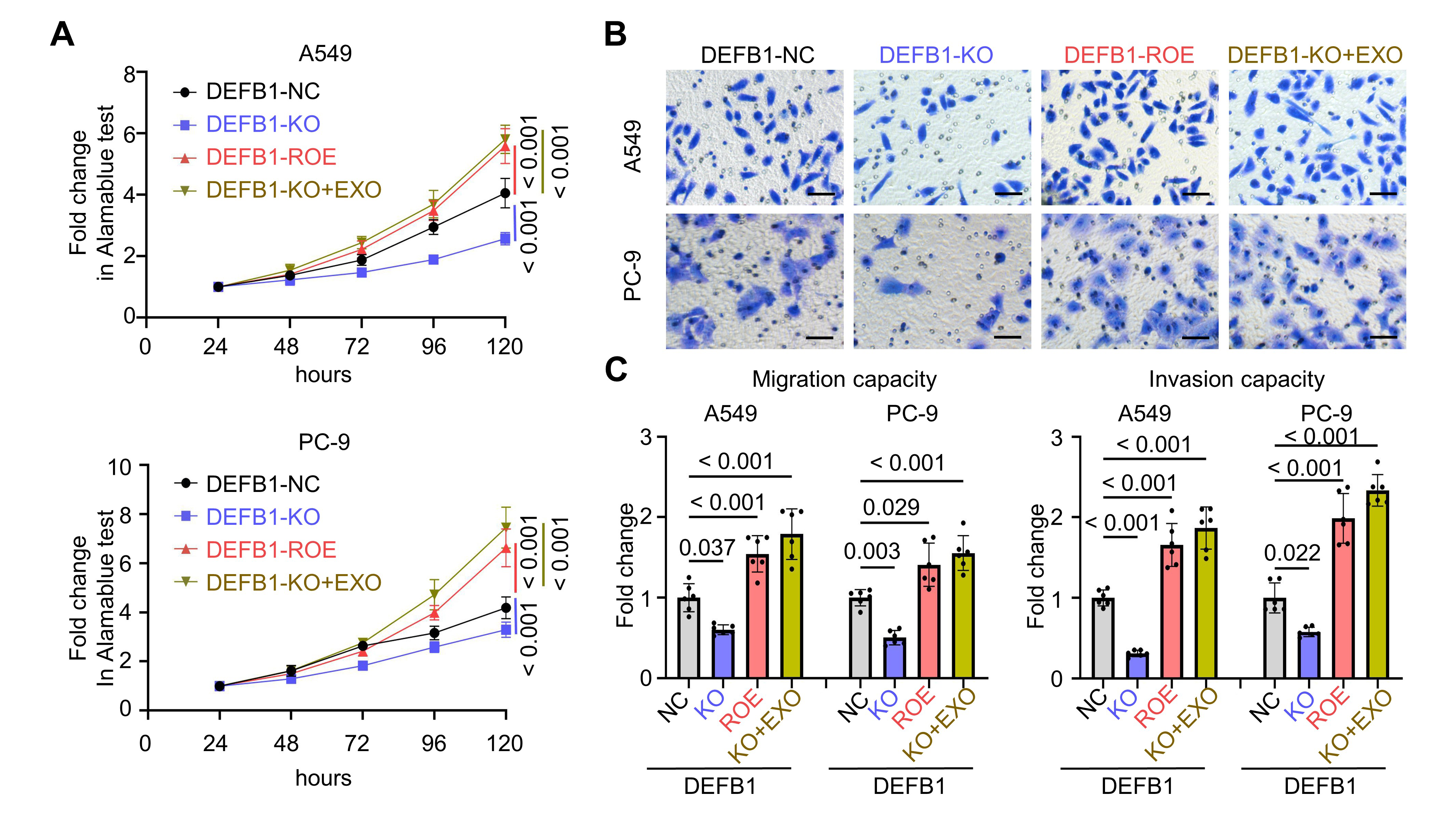


# Supplementary figure 2. DEFB1 enhances lung adenocarcinoma proliferation, migration, and invasion *in vitro* and tumorigenesis *in vivo*

(A) Alama blue assay for the proliferation of A549/PC-9 DEFB1-NC/KO/ROE/KO+EXO cells; (B) Transwell assay for the migration capacity of A549/PC-9 DEFB1-NC/KO/ROE/KO+EXO cells (scale bar: 100 μm); (C) Statistical Analysis of the migration and invasion capacities of A549/PC-9 DEFB1-NC/KO/ROE/KO+EXO cells. Statistical analysis: (A) Two-way ANOVA test (*n* = 6) and Bonferroni correction; (B) One-way Annova and Bonferroni correction.


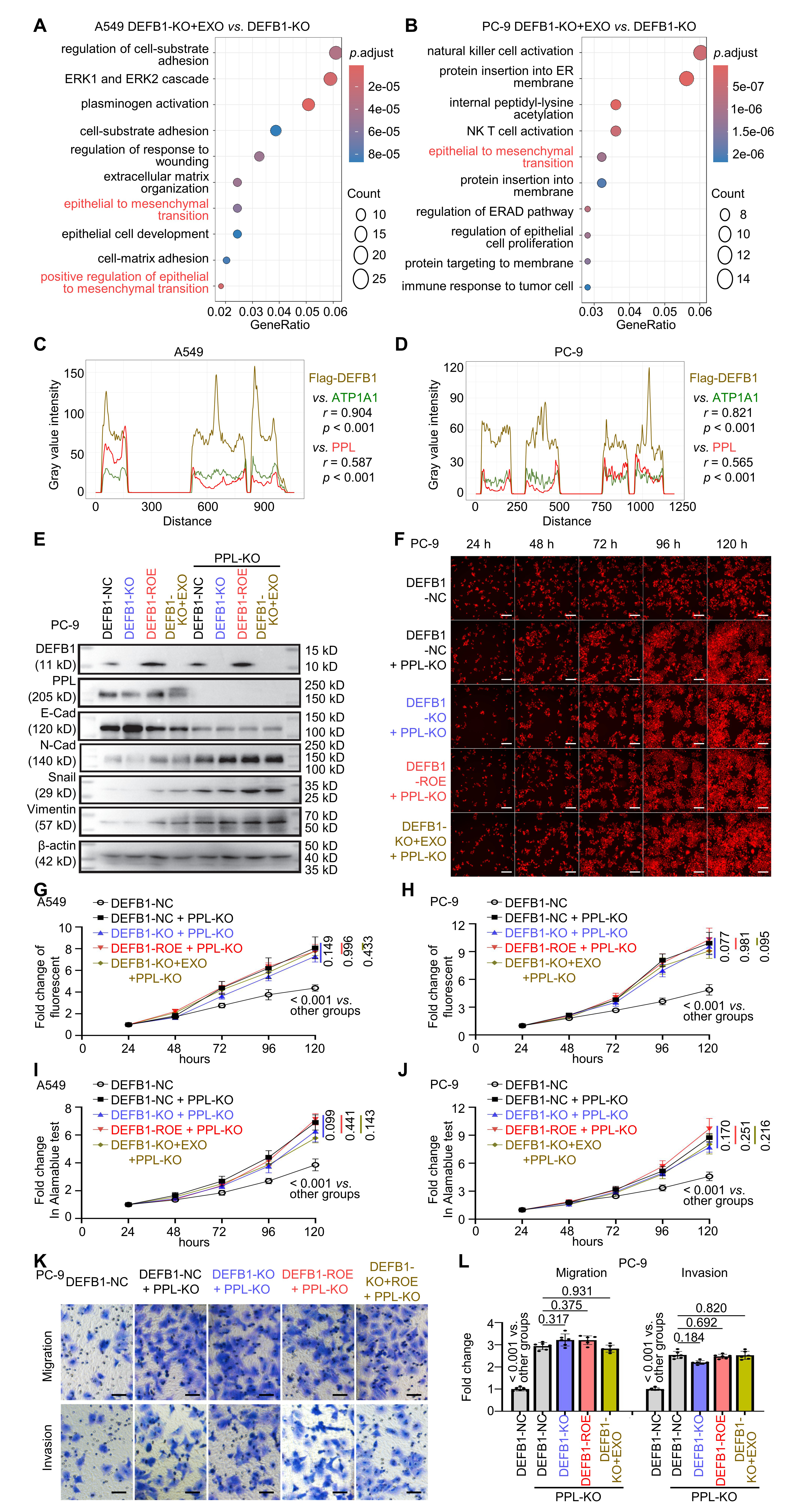


# Supplementary figure 3. DEFB1 interacts with Periplakin (PPL) to promote epithelial-to-mesenchymal transition (EMT) and proliferation in lung adenocarcinoma cells

(A-B) Gene Ontology (GO) biological process enrichment analysis of the differentially expressed genes between A549/PC-9 DEFB1-KO+EXO cells and DEFB1-KO cells; (C-D) Line-scan plots of fluorescence intensity along the dashed lines indicated in Fig. 3E and Pearson’s correlation analyses between Flag-DEFB1 and ATP1A1 or PPL; (E) Western blot detection of the expression of EMT-related proteins in PC-9 DEFB1-NC/KO/ROE/KO+EXO cells with or without PPL knockout (PPL-KO); (F) High-content imaging (HCI) for the proliferation of PC-9 DEFB1-NC, and DEFB1-NC/KO/ROE/KO+EXO cells with PPL-KO (scale bar: 200 μm); (G-J) Proliferation statistics of A549/PC-9 DEFB1-NC, and DEFB1-NC/KO/ROE/KO+EXO cells with PPL-KO using high-content imaging and Alama blue assays; (K-L) Transwell assays of the migration and invasion capacities of PC-9 DEFB1-NC, and DEFB1-NC/KO/ROE/KO+EXO cells with PPL-KO (scale bar: 100 μm). Statistical analysis: (C-D) Pearson’s correlation analyses; (G-J) Two-way ANOVA (*n* = 6) and Bonferroni correction; (L) One-way ANOVA test and Bonferroni correction.


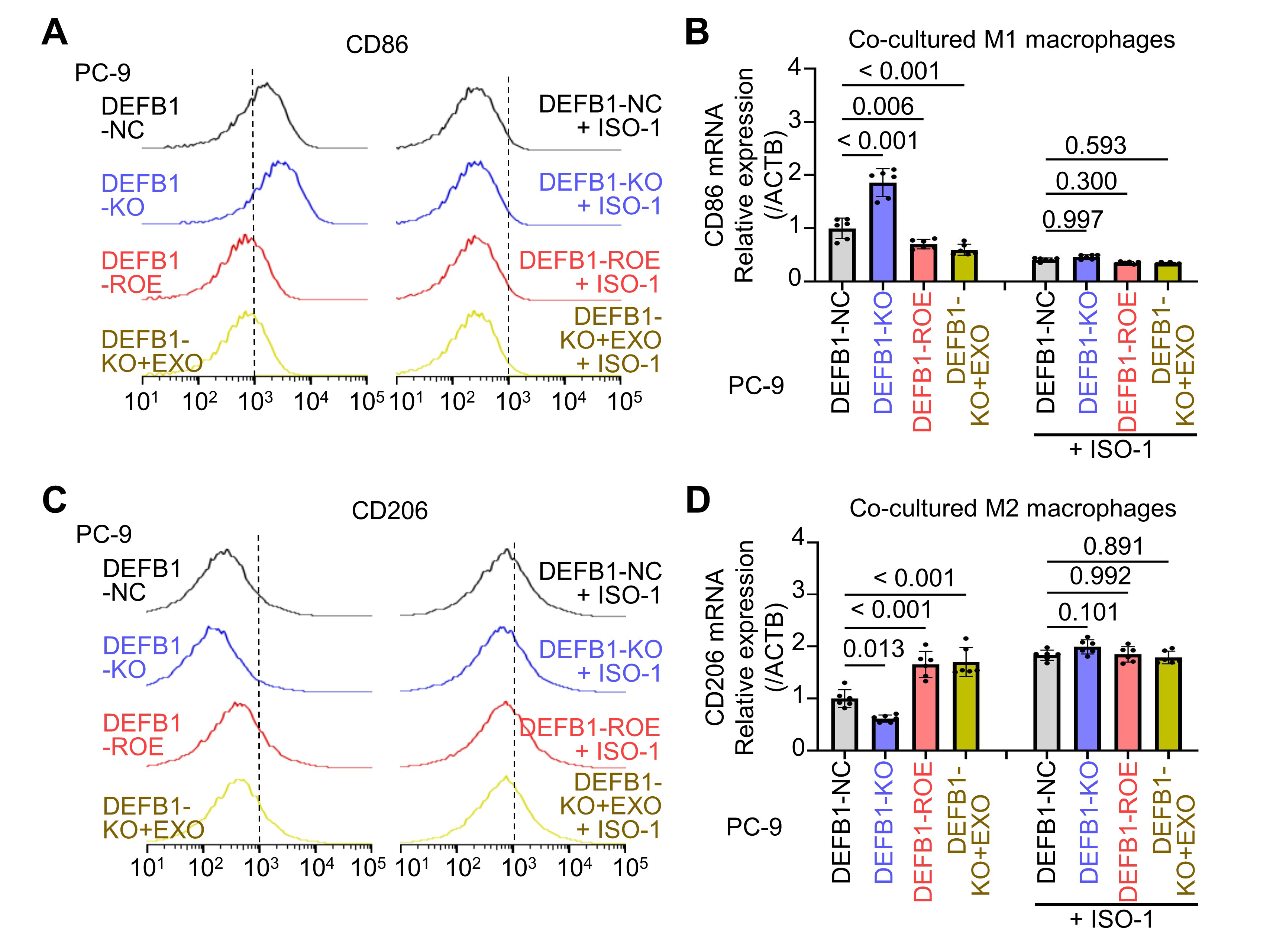


# Supplementary figure 4. DEFB1 interacts with Macrophage Migration Inhibitory Factor (MIF) to promote M2 polarization of macrophages *in vitro* and *in vivo*

(A-B) Flow Cytometry and qRT-PCR for evaluating the effects of DEFB1 and/or MIF inhibitor (ISO-1) on M1 polarization of THP-1 M0 cells co-cultured with PC-9 cells; (C-D) Flow Cytometry and qRT-PCR for evaluating the effects of DEFB1 and/or ISO-1 on M2 polarization of THP-1 M0 cells co-cultured with PC-9 cells. Statistical analysis: (B, D) One-way ANOVA test and Bonferroni correction.


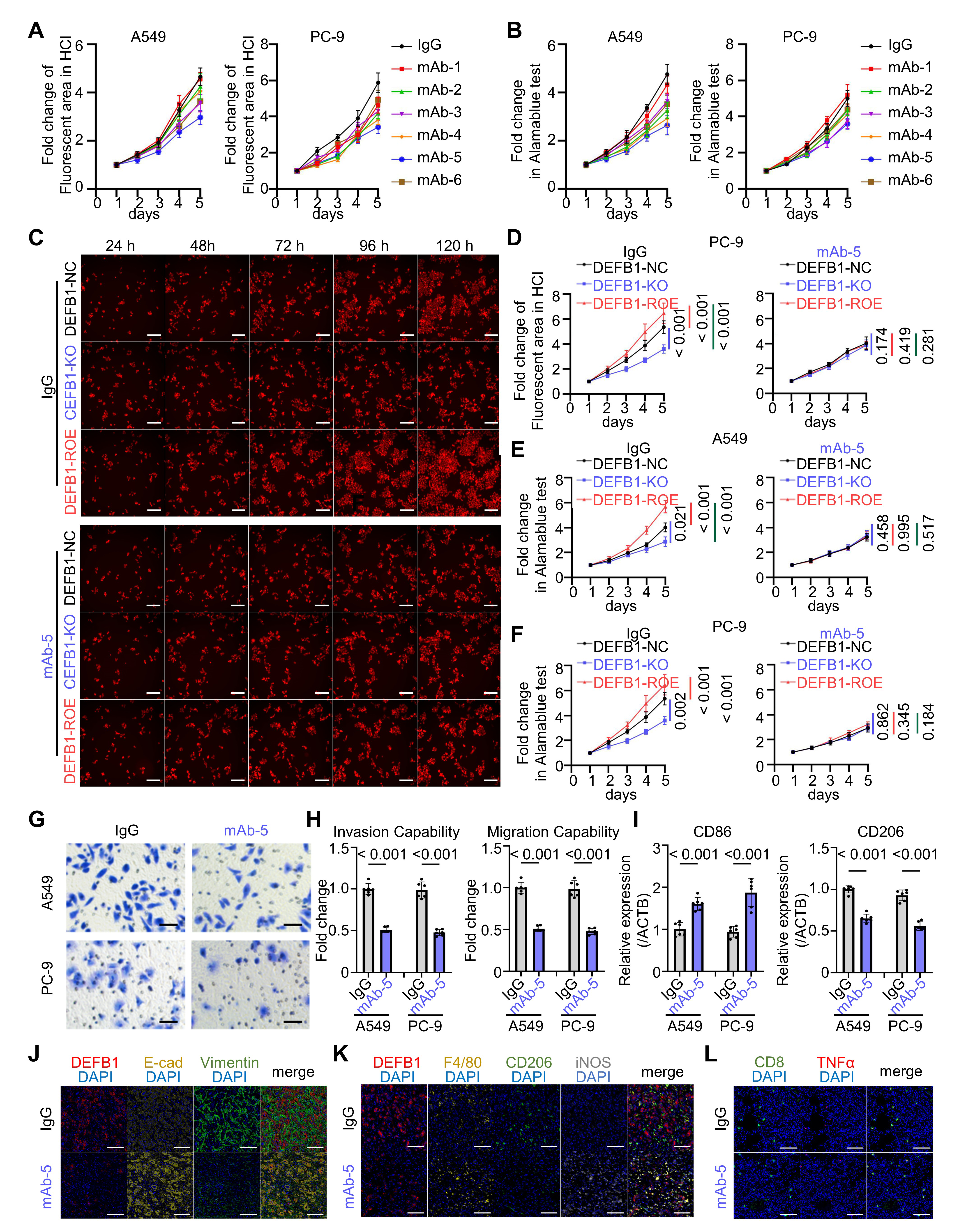


# Supplementary figure 5. Anti-DEFB1 monoclonal antibody as a promising therapeutic agent against lung adenocarcinoma

(A-B) High-content imaging and Alama blue assays for the proliferation of A549 and PC-9 cells treated with 1 μg/mL IgG or anti-DEFB1 monoantibodies; (C-D) High-content imaging (HCI) and the statistical analysis for the proliferation of PC-9 DEFB1-NC/KO/ROE cells treated with 1 μg/mL IgG or anti-DEFB1 monoantibody mAb-5 (scale bar: 200 μm); (E-F) Alama blue assays for the proliferation of A549/PC-9 DEFB1-NC/KO/ROE cells treated with 1 μg/mL IgG or anti-DEFB1 monoantibody mAb-5; (G) Transwell assay for the migration capacity of A549/PC-9 cells treated with 1 μg/mL IgG or mAb-5 (scale bar: 100 μm); (H) Statistical analysis of the Transwell assay results evaluating the invasion and migration capacities of A549/PC-9 cells treated with 1 μg/mL IgG or mAb-5; (I) qRT-PCR for evaluating the M1/M2 polarization of THP-1 M0 cells co-cultured with A549/PC-9 cells treated with 1 μg/mL IgG or mAb-5; (J-K) Epithelial-mesenchymal transition (EMT) and macrophage infiltrating statuses in nude-mouse xenografts treated with IgG or mAb-5 (scale bar: 200 μm); (L) CD8+ T cells infiltrating and function statuses in spontaneous lung tumor mice model treated with IgG or mAb-5 (scale bar: 200 μm). Statistical analysis: (A, B, D-F) Two-way ANOVA test (*n* = 6) and Bonferroni correction; (H-I) Student's *t*-test.
