# Supplementary figures and images for: Unveiling DEFB1 as a novel driver and promising therapeutic target in lung adenocarcinoma
Source: Cell Death Dis. 2026 Apr 20;17(1):519. doi: 10.1038/s41419-026-08748-4 (PMC13223247; doi:10.1038/s41419-026-08748-4)

**Fig. 1G**


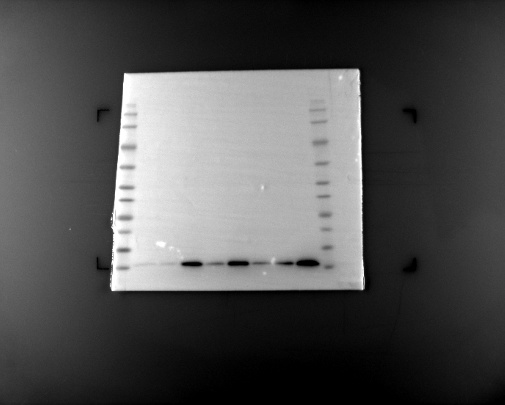

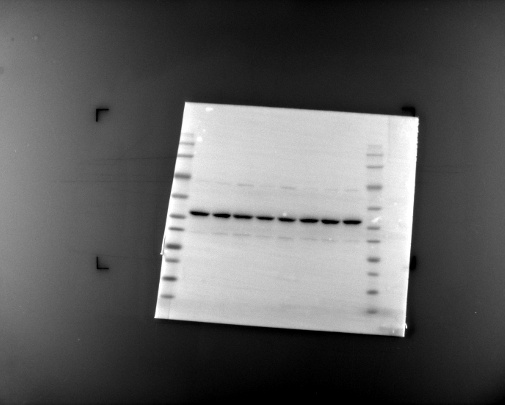


**Fig. 1H**


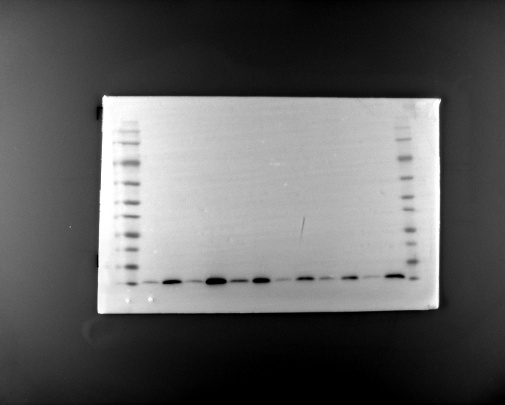

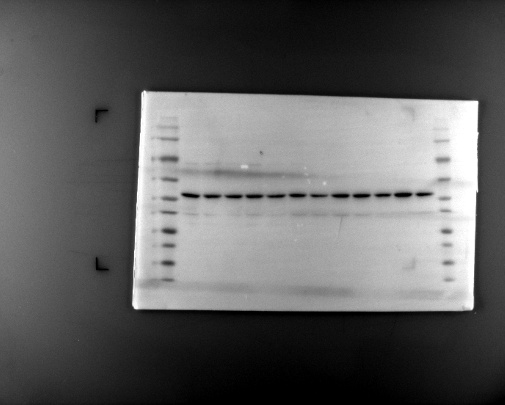


**Fig. 2A**


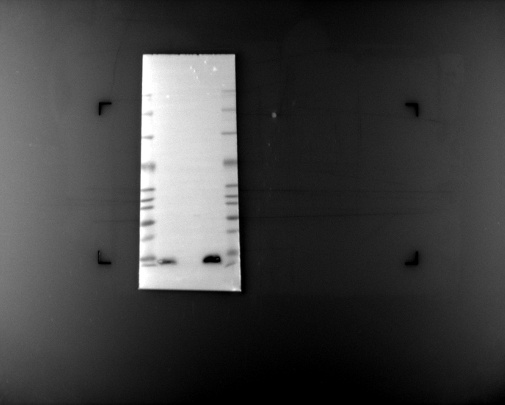

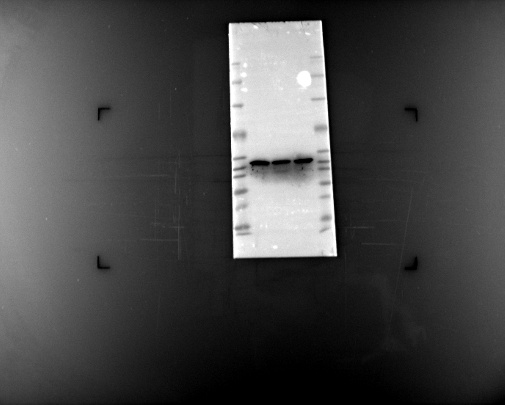

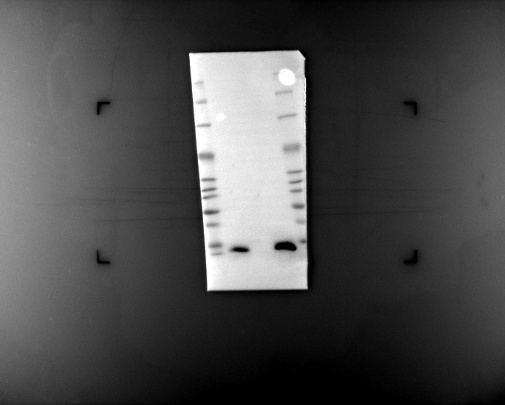

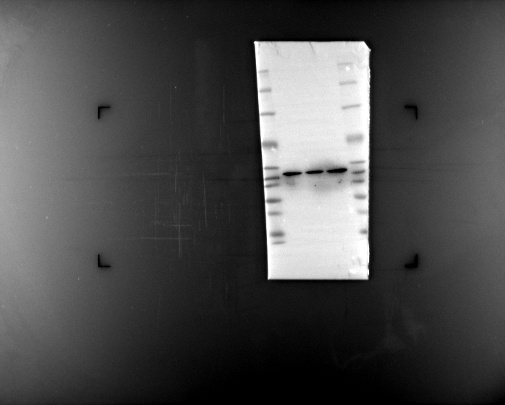


**Fig. 3C**


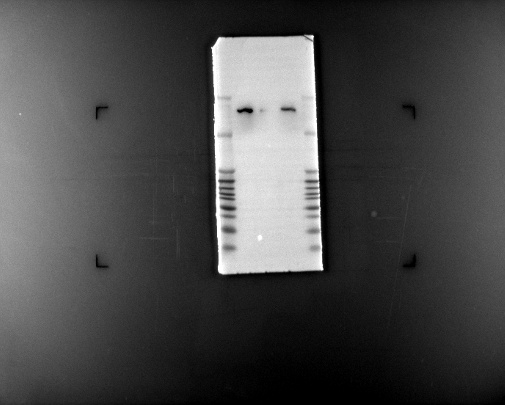

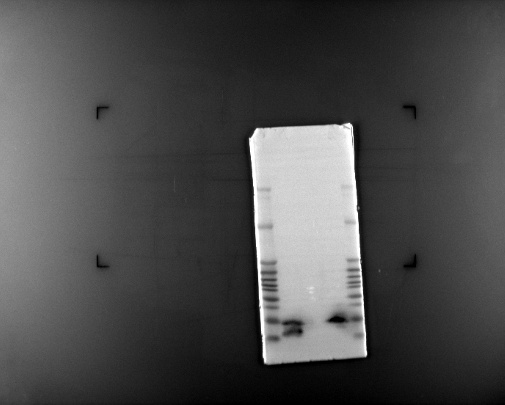


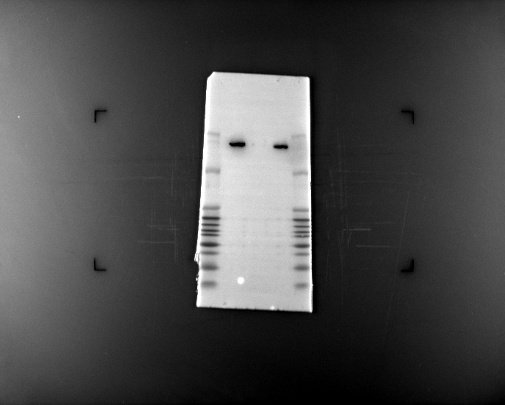

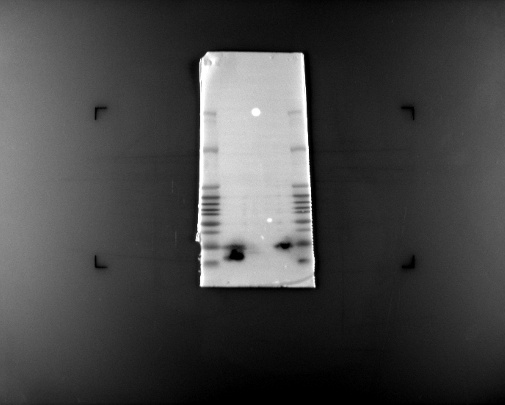


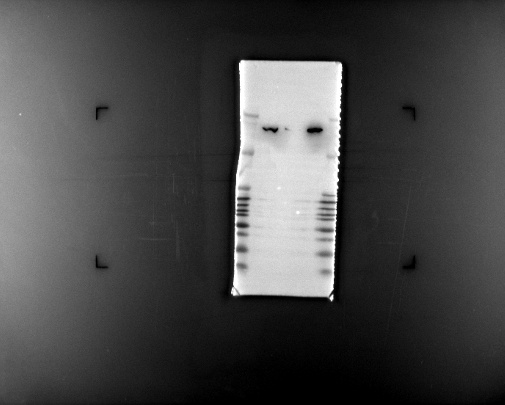

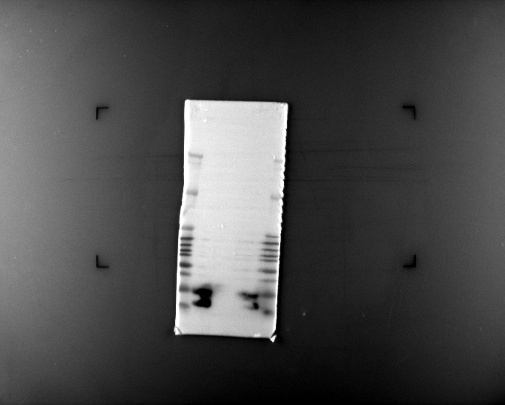


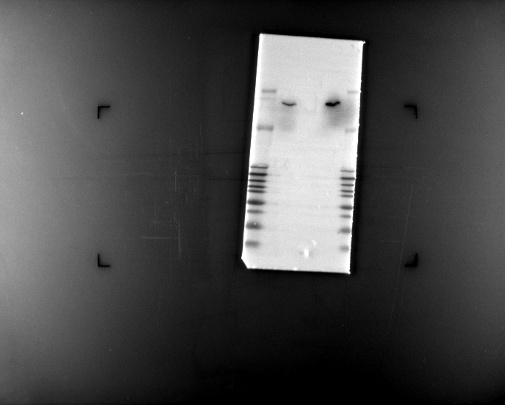

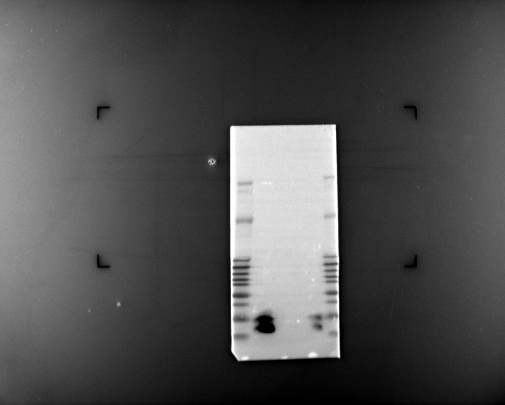


**Fig. 3G**


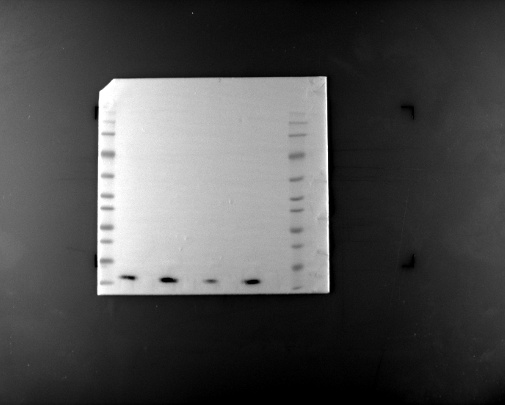

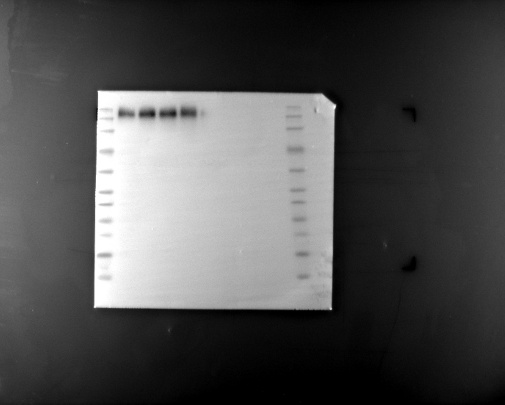

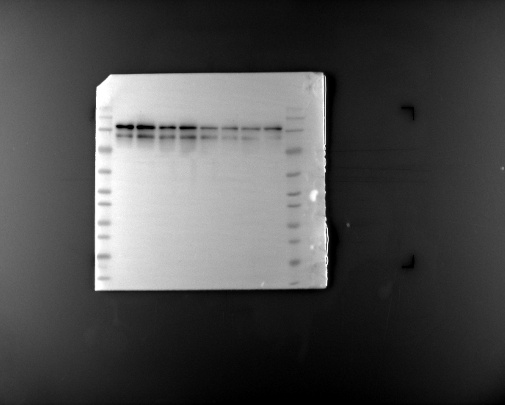

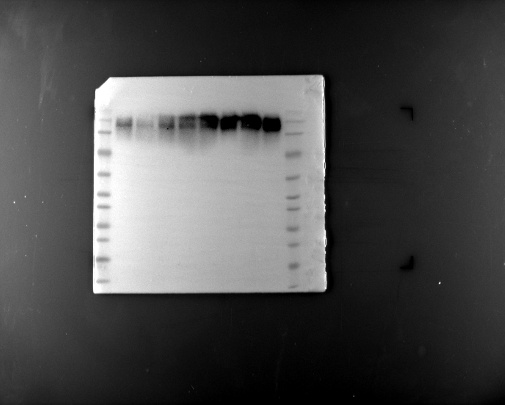

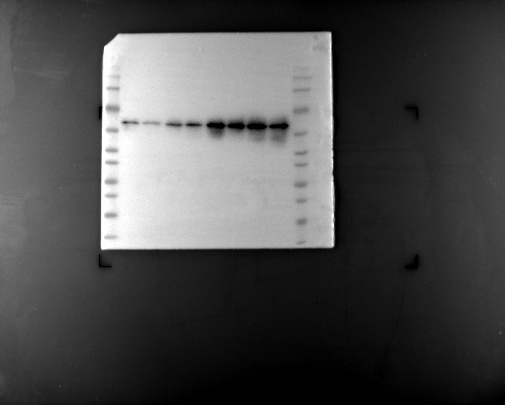

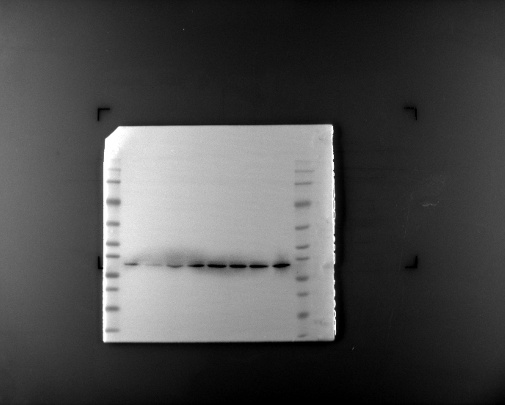

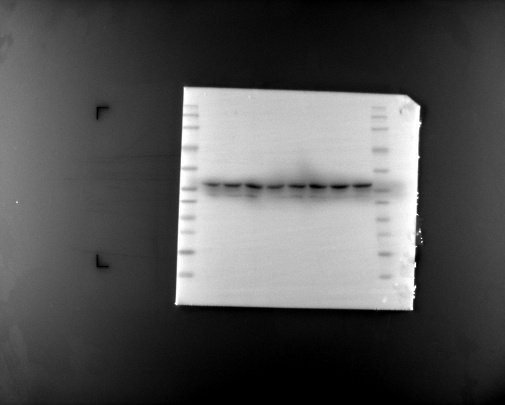


**Fig. 4B**


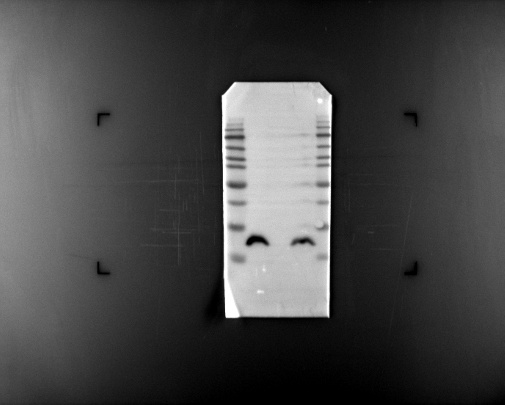

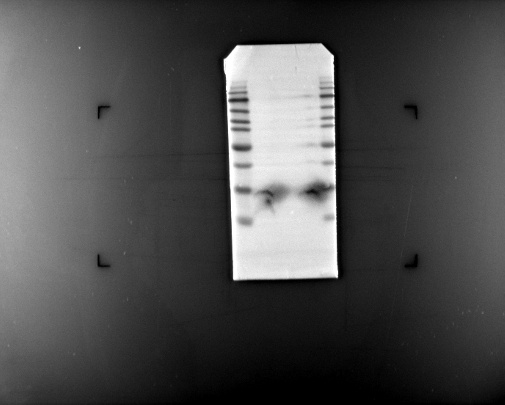

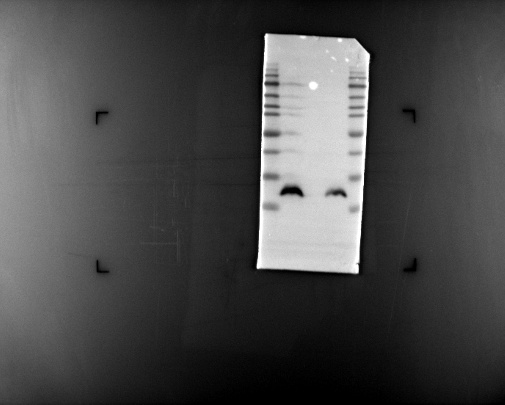

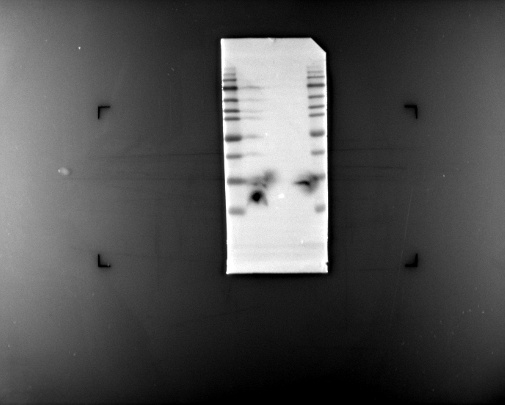

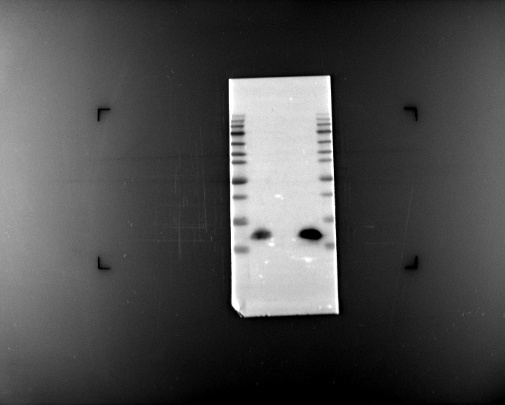

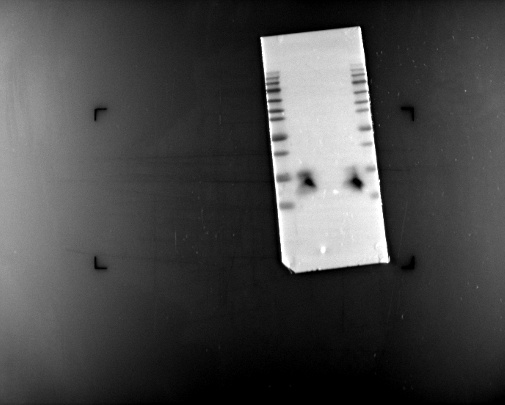


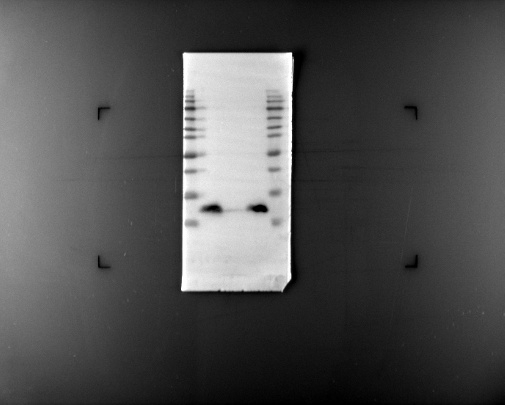

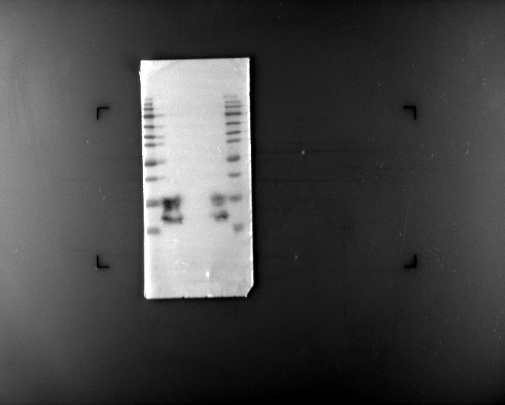


**Fig. 5D**


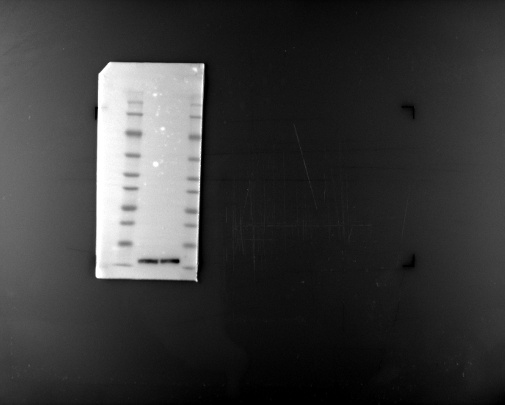

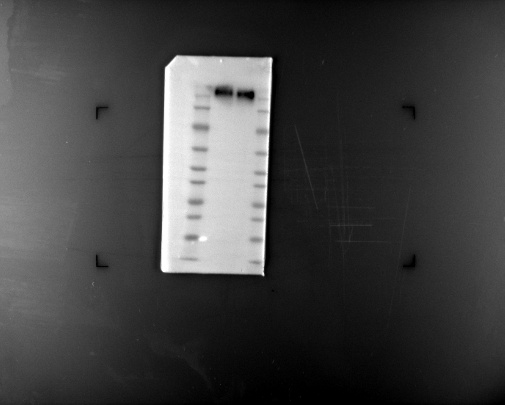


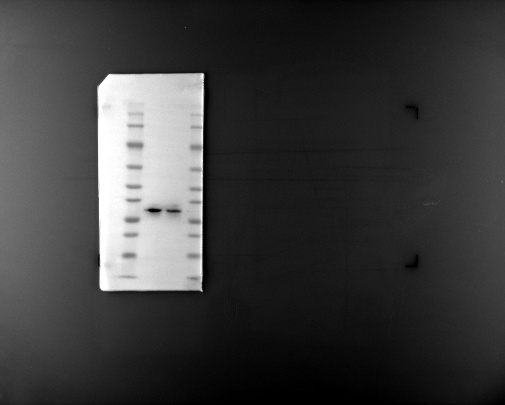

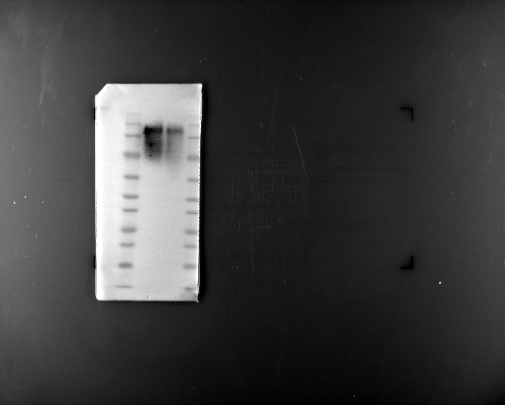

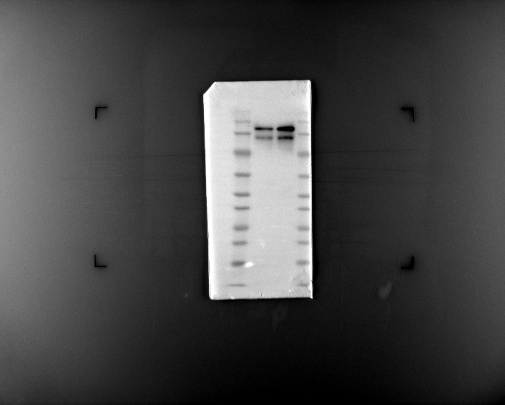

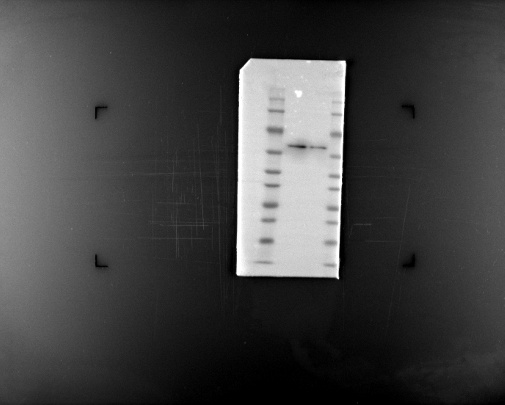


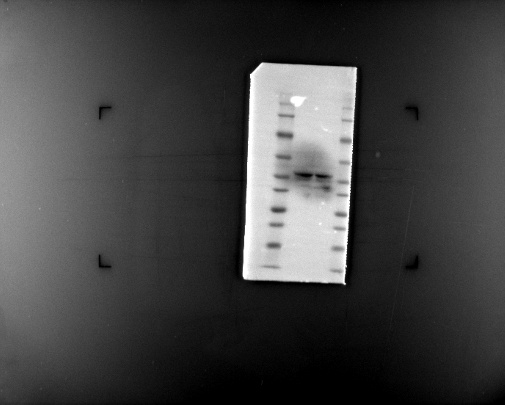


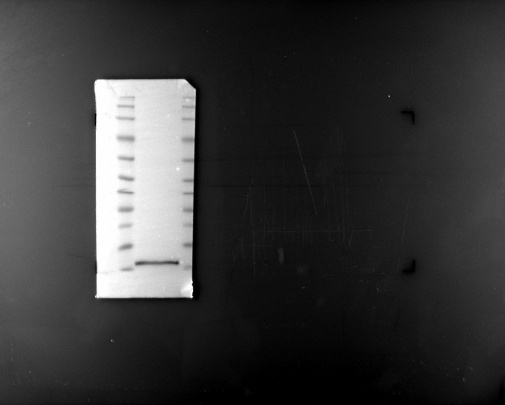

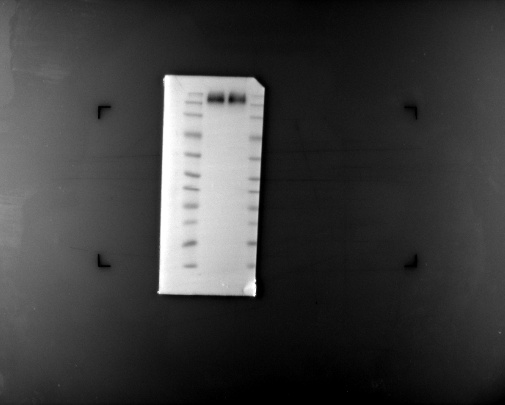

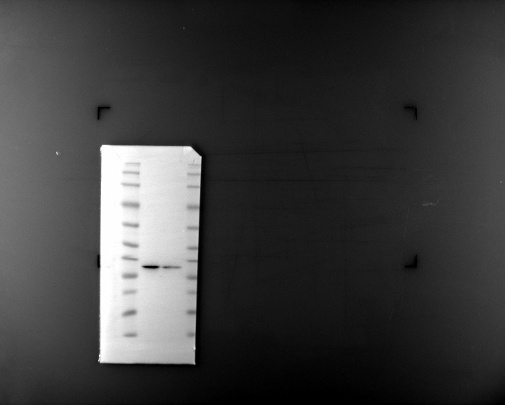

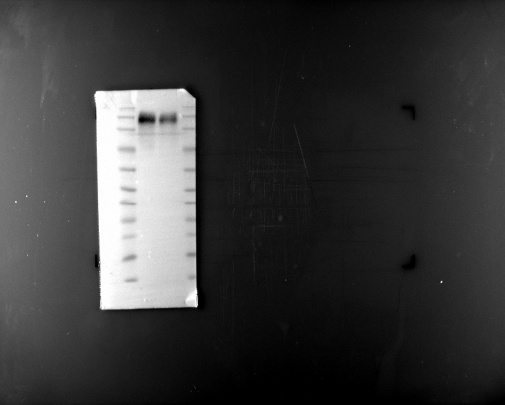

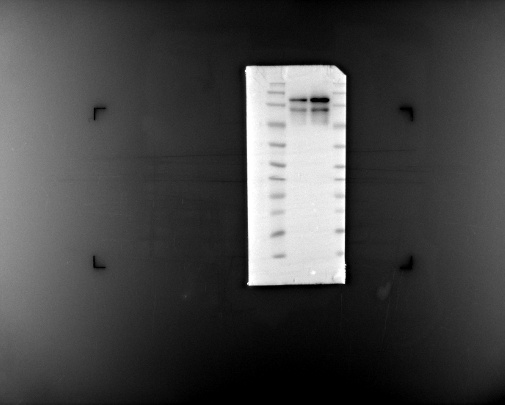

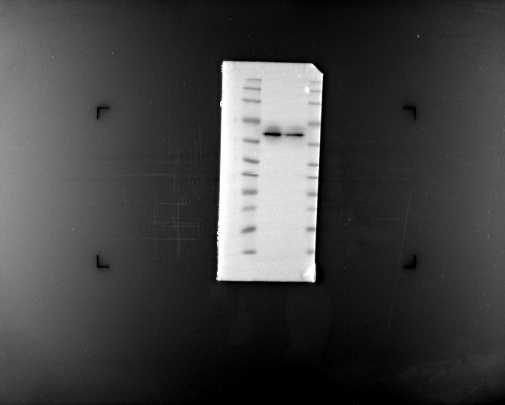

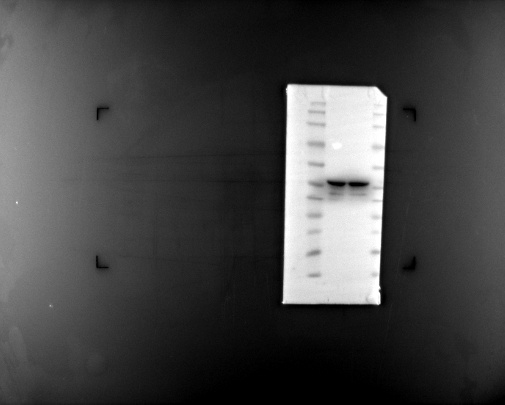


**Fig. S3C**


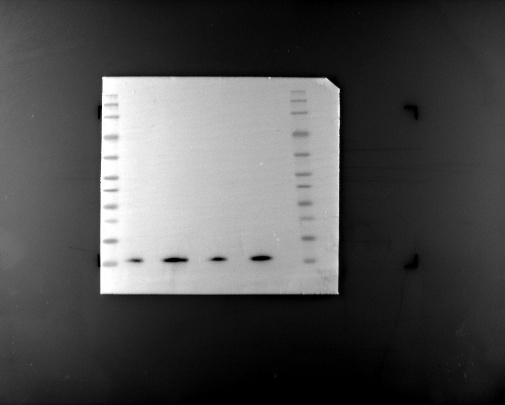

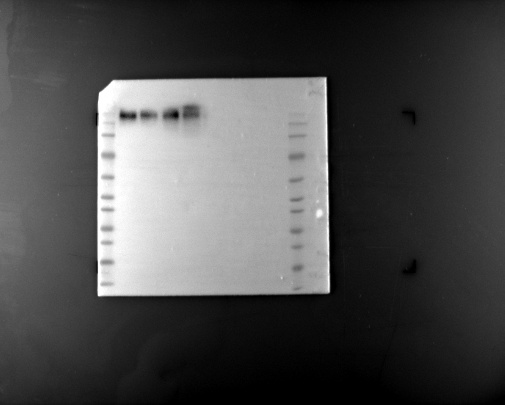

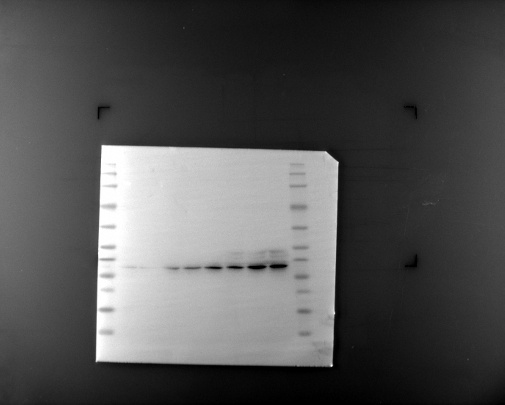

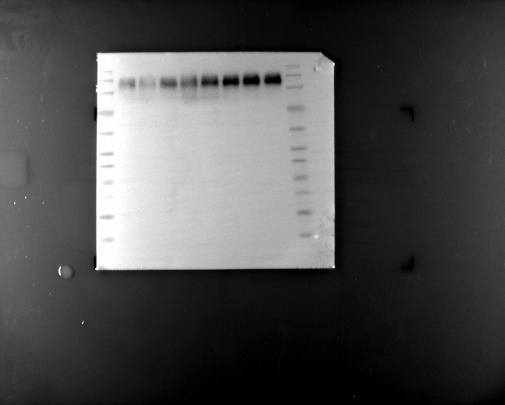

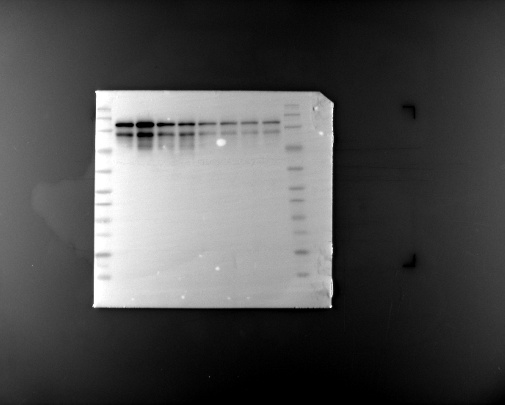

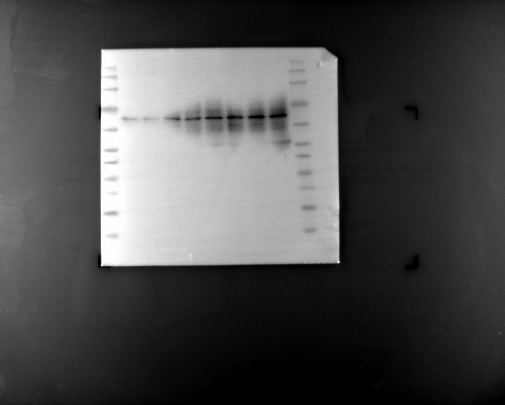

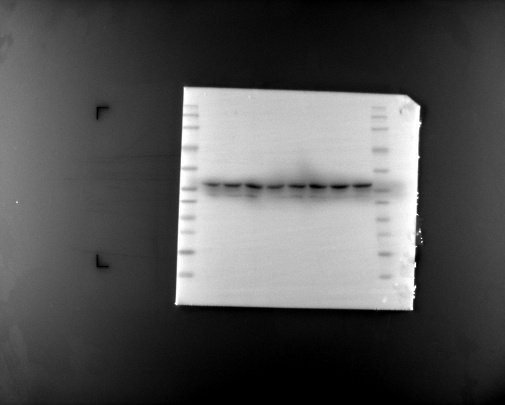

Supplement: Supplementary file 3 — Original Western blots [file 41419_2026_8748_MOESM3_ESM.docx]
